# Integrative Bioinformatics and Exploratory Experimental Validation of Expression Changes in miR-214-3p, miR-1b, miR-133b-3p, and Directionally Concordant mRNAs in a Rat Model of Acute Myocardial Infarction

**DOI:** 10.3390/medicina62101860

**Published:** 2026-09-25

**Authors:** Bilge Eren Yamasan, Mustafa Yıldız, Selçuk Korkmaz

**Affiliations:** 1 Department of Biophysics, Faculty of Medicine, Trakya University, 22030 Edirne, Türkiye; berenyamasan@trakya.edu.tr (B.E.Y.); mustafa.yildiz@trakya.edu.tr (M.Y.); 2 Department of Biostatistics, Faculty of Medicine, Trakya University, 22030 Edirne, Türkiye

**Keywords:** acute myocardial infarction, miRNA–mRNA directional concordance, cardiac remodeling, fibrosis, biomarkers

## Abstract

*Background and Objectives*: Acute myocardial infarction (AMI) induces inflammatory, cell-death, and fibrotic responses. MicroRNAs modulate these processes by regulating specific mRNA targets, but the contribution of individual miRNA–mRNA axes remains incompletely defined. *Materials and Methods*: Transcriptomic datasets GSE35088 (mRNA) and GSE95855 (miRNA) were analyzed to identify differentially expressed mRNAs and miRNAs. Pooled predicted targets of upregulated miRNAs were intersected with upregulated mRNAs, and those of downregulated miRNAs with downregulated mRNAs, to prioritize directionally concordant candidate sets. The canonical inverse-direction intersections were computed for comparison. An isoproterenol-induced model of myocardial injury in male Wistar rats, with injury verified by creatine kinase-MB and troponin-I, was used for exploratory RT-qPCR. *Results*: The analysis yielded 143 differentially expressed mRNAs and 35 miRNAs. The same-direction intersection prioritized Fbn1, Col3a1, Hamp, and Cyp2e1, grouped by direction with the most strongly changed miRNAs: upregulated miR-214-3p and downregulated miR-1b and miR-133b-3p. Among these molecules, only Fbn1 has predicted interactions with these miRNAs in the multiMiR output, with miR-214-3p in the same direction and miR-133b-3p in the inverse direction. The canonical intersection identified 28 upregulated mRNAs as predicted targets of downregulated miRNAs, twelve of which, including Col3a1, are targets of the miR-29 family. RT-qPCR showed directionally concordant, statistically supported changes for miR-214-3p, miR-1b, miR-133b-3p, Col3a1, and Cyp2e1, reported as fold changes with 95% confidence intervals. Hamp and Fbn1 were not statistically supported, with intervals wide enough to include decreases as well as several-fold increases. *Conclusions*: The candidate sets reflect directional concordance, not demonstrated miRNA–target regulation. The upregulated set (miR-214-3p, Col3a1) and the downregulated set (miR-1b, miR-133b-3p, Cyp2e1) comprise molecules previously implicated in fibrotic and oxidative remodeling, while the canonical analysis nominates the miR-29 family for future testing. These molecules warrant evaluation in circulating samples and larger cohorts.

## 1. Introduction

Acute myocardial infarction (AMI) is a leading cause of morbidity and mortality worldwide, caused by rupture of atherosclerotic plaques leading to sudden coronary occlusion and prolonged ischemia [1]. The resulting injury causes irreversible cardiomyocyte loss, triggers inflammation, and leads to fibrotic scar formation. Post-infarction fibrosis remodels the extracellular matrix, drives adverse cardiac remodeling, and contributes to heart failure [2]. Accordingly, both the timely clinical management of AMI and the understanding of its molecular mechanisms are critical to improving patient outcomes.

MicroRNAs (miRNAs) have emerged as important post-transcriptional regulators in cardiovascular biology and disease. These short non-coding RNAs (~19–24 nucleotides) typically bind to the 3′ untranslated regions (3′UTRs) of target mRNAs, causing transcript destabilization or translational repression [3]. More than 2000 miRNAs have been identified in the human genome [4], and collectively they are estimated to regulate roughly 60% of protein-coding genes [5]. Through this extensive scope, miRNAs influence diverse processes ranging from cardiac development and electrophysiology to vascular homeostasis, metabolism, and disease pathogenesis [6]. Numerous studies have implicated miRNAs in AMI-related processes, including oxidative stress, apoptosis, inflammation, fibrosis, and remodeling [7,8,9,10,11,12,13,14]. A recent review has further synthesized global perspectives on these regulatory mechanisms [15]. For example, miR-21 has been shown to modulate p38 mitogen-activated protein kinase (MAPK) and nuclear factor kappa-light-chain-enhancer of activated B cells (NF-κB) signaling pathways that limit inflammatory injury [16,17], while miR-143-3p promotes fibrosis through activation of MAPK pathways in fibroblasts [18]. These findings underscore the critical role of miRNA–mRNA interactions in orchestrating the molecular response to ischemic injury.

Beyond their mechanistic importance, circulating miRNAs have attracted attention as candidate biomarkers for AMI. Cardiac troponins remain the reference standard for diagnosis, and conventional assays show delayed elevation after symptom onset and may lack specificity in some settings [19]. Cardiac-enriched miRNAs are released into the circulation rapidly after ischemic injury [20], although whether they add diagnostic information to contemporary high-sensitivity troponin assays remains unresolved. Several miRNAs, including miR-1, miR-133a/b, miR-208a/b, and miR-499, are consistently elevated in plasma after infarction and have been evaluated for diagnostic and prognostic purposes [21,22,23,24]. Circulating miR-1 has been associated with subsequent left ventricular remodeling after ST-segment elevation myocardial infarction (STEMI) [25], and combinations such as miR-146a-5p with miR-21 have been shown to predict adverse remodeling [26]. These observations highlight the potential of miRNA signatures to complement existing biomarkers and refine risk stratification in AMI [27].

Despite recent advances, major gaps persist in understanding how miRNAs regulate the molecular landscape of AMI through their mRNA targets. Most studies have profiled miRNAs or mRNAs separately, limiting insight into coordinated post-transcriptional programs. Integrative approaches are emerging, but few have systematically defined reproducible miRNA–mRNA relationships linked to fibrosis, inflammation, and adverse remodeling. For example, Wang and Cao [2] identified 19 differentially expressed miRNAs and over 1000 putative mRNA targets in AMI patient blood, implicating immune and stress pathways such as chemokine signaling, Janus kinase–signal transducer and activator of transcription (JAK–STAT), and MAPK cascades. While informative, key regulatory axes relevant to myocardial injury and remodeling remain incompletely characterized, and experimental validation is often lacking. Recent multi-omics frameworks for cardiac fibrosis [28] and machine-learning-based AMI miRNA biomarker panels [29] further illustrate the breadth of computational AMI research, yet specific miRNA–mRNA regulatory axes coupling fibrotic remodeling with oxidative injury have not been systematically defined.

To address these gaps, we integrated bioinformatic analyses of public transcriptomic datasets with experimental validation in a rat model of isoproterenol-induced myocardial injury. Our goals were to identify differentially expressed miRNAs and mRNAs, map predicted miRNA–mRNA interactions and directionally concordant expression changes associated with adverse remodeling, and examine the prioritized candidate sets in vivo. Linking computational predictions with experimental data provides an integrated view of post-transcriptional changes in AMI and highlights individual molecules with potential biomarker and therapeutic relevance. Because the discovery datasets and the validation cohort differ in injury model, sampling time, strain, the region of the left ventricle sampled, and, for the mRNA dataset, sex, agreement between them indicates directionally concordant expression changes across the contexts examined rather than conservation across acute myocardial infarction generally. In contrast to prior studies that have profiled miRNAs or mRNAs in isolation, we integrate both molecular layers, prioritize a small set of directionally concordant miRNAs and mRNAs as candidate sets rather than as inferred regulatory pairs for exploratory in vivo examination, and report the canonical inverse-direction intersections alongside them. This approach yields an integrated description of a downregulated candidate set of oxidative-stress-associated molecules (miR-1b, miR-133b-3p and Cyp2e1) and an upregulated candidate set of fibrosis-associated molecules (miR-214-3p and Col3a1) in the post-infarction myocardium.

## 2. Materials and Methods

### 2.1. Datasets

To identify differentially expressed mRNAs and miRNAs in AMI, we analyzed two rat microarray datasets from GEO. The mRNA dataset GSE35088 (Illumina RatRef-12 v1.0 Expression BeadChip, GPL6101; Illumina, Inc., San Diego, CA, USA) profiles non-infarcted (remote) left ventricular tissue from female Sprague-Dawley rats four weeks after LAD ligation and includes sham (*n* = 5), AMI (*n* = 5), and T4-treated (*n* = 5) groups, of which only sham and AMI were used to isolate MI-specific changes [30]. The miRNA dataset GSE95855 (Agilent-070154 Rat miRNA Microarray, GPL22760; Agilent Technologies, Inc., Santa Clara, CA, USA) contains left ventricular profiles, for which the sampled region is not further specified in the source study, from male Sprague-Dawley rats four weeks after LAD ligation-induced AMI (AMI *n* = 3, sham *n* = 3) [31]. Both were downloaded with the GEOquery package (version 2.80.0) in R [32]. The two discovery datasets were analyzed together because they share strain, injury model, and a four-week post-ligation sampling point, but they differ in sex, with GSE35088 having profiled female and GSE95855 male animals. The direction matching described in Section 2.3 therefore relates mRNA changes measured in female rats to miRNA changes measured in male rats. The validation cohort described below differs from both discovery datasets in injury model, sampling time, and strain, comprising male Wistar rats sampled at the end of a two-day isoproterenol protocol, and whole left ventricular tissue was used for validation because diffuse catecholamine injury does not produce the distinct infarct and remote zones that follow coronary ligation. Because injury model, sampling time, strain, the region of the left ventricle sampled and, for the mRNA dataset, sex all differ simultaneously, agreement or disagreement between the discovery and validation stages cannot be attributed to any one of them.

### 2.2. Differential Expression Analysis

For GSE35088, platform-provided log2 values were used, whereas for GSE95855 raw Agilent signals were log2-transformed. Both datasets underwent quantile normalization and differential expression analysis with limma [33] (standard lmFit, contrasts.fit, eBayes, and topTable workflow with FDR-adjusted *p*-values), comparing AMI vs. sham. Multiple testing was controlled by Benjamini–Hochberg. mRNAs and miRNAs were deemed differentially expressed at adjusted *p* < 0.05 and |log2FC| ≥ 0.5, thresholds that align with common practice for whole-transcriptome arrays in cardiac tissue. Among the differentially expressed miRNAs, the three with |log2FC| ≥ 2.5 (miR-214-3p, miR-1b and miR-133b-3p) were highlighted as the most strongly altered and carried forward to RT-qPCR. This stricter threshold served only for this selection and not to define the miRNA set used for target mapping. Predicted miRNA–mRNA interactions from multiMiR were overlapped with differentially expressed genes, separately for each direction of change, with target sets pooled across all differentially expressed miRNAs of that direction (Section 2.3). Candidates for RT-qPCR were then prioritized among the overlapping mRNAs using four criteria considered in combination. First, fold-change magnitude was assessed using |log2FC|, and a filter of |log2FC| > 0.9 was applied to the intersected genes after the intersections (the counts in Section 3.3 are pre-filter), retaining Crlf1_predicted (0.94), Fbn1 (0.95), Col3a1 (0.95) and Hamp (1.33) among upregulated and Cyp2e1 (−0.905) among downregulated genes. Crlf1_predicted carries the legacy predicted-gene annotation of the RatRef-12 platform and was not pursued. Second, statistical significance was evaluated using the Benjamini–Hochberg-adjusted *p*-value. Third, myocardial expression abundance was assessed as the mean log2-intensity of each candidate transcript in AMI samples from the discovery datasets. Higher-abundance transcripts were preferred to support reliable RT-qPCR detection in cardiac tissue. Fourth, functional relevance was prioritized for genes and miRNAs with established or biologically plausible roles in cardiac injury, fibrosis, oxidative stress, inflammation, or post-infarction remodeling, as supported by the published cardiac biology literature [12,15]. A selected subset was examined by reverse transcription quantitative polymerase chain reaction (RT-qPCR) in the rat isoproterenol model. Differential expression results were visualized as volcano plots using the EnhancedVolcano (version 1.30.0) R package.

### 2.3. Mapping DE miRNAs to Predicted mRNA Targets

Putative targets of differentially expressed miRNAs were retrieved using the multiMiR database [34], which aggregates experimentally validated and predicted miRNA–mRNA interactions. Predicted interactions were used (multiMiR was queried with table = “predicted” and species = “rno”). Target gene lists obtained via multiMiR were then intersected with the set of differentially expressed mRNAs from GSE35088, separately by direction of change, so that predicted targets of upregulated miRNAs were intersected with upregulated mRNAs, and predicted targets of downregulated miRNAs with downregulated mRNAs. In each intersection, the predicted-target sets were pooled across all upregulated or all downregulated miRNAs, respectively, so that membership of an mRNA in an intersection indicates a predicted interaction with at least one differentially expressed miRNA of that direction and not necessarily with the miRNAs highlighted in Section 3.1. Because the same-direction intersections select concordant changes by construction, the resulting groups are treated as directionally concordant candidate sets prioritized for exploratory validation rather than as regulatory axes. Directional concordance here denotes agreement in the direction of differential expression between the two independent datasets, not correlation of expression across samples, which could not be computed because the datasets contain different animals.

multiMiR database version 2.4 (updated 28 August 2024) was queried with the package’s default prediction settings, which retain the top 20% of predictions in each database and, where a database distinguishes them, conserved target sites. Of the eight prediction resources in multiMiR, four (ElMMo, MicroCosm, miRanda and miRDB) contain rat predictions. Throughout, “predicted target” refers to this queried output, and the absence of a prediction in it does not exclude predictions in resources without rat coverage. For comparison, and to allow canonical inverse relationships to be assessed, the opposite-direction intersections were also computed with the identical query and gene sets, so that predicted targets of upregulated miRNAs were intersected with downregulated mRNAs, and predicted targets of downregulated miRNAs with upregulated mRNAs. For each intersection, and for each miRNA separately, the overlap expected under independence was estimated with a one-sided Fisher exact test. All probes were eligible for differential-expression selection, and the universe was the *N* = 21,674 unique gene symbols among the 22,523 tested probes. The 117 probes without a symbol could not be matched to a prediction, and none of them were differentially expressed. Predicted targets were restricted to that universe, leaving 2143 of the 5357 predicted targets of upregulated miRNAs and 2120 of the 5222 targets of downregulated miRNAs. The expected overlap is n × m/N for n differentially expressed genes and m array-represented targets. The four pooled tests are reported unadjusted, with Bonferroni correction across the four tests also given. The per-miRNA tests are Benjamini–Hochberg adjusted once across all 35 miRNAs, each tested against the oppositely regulated genes. Gene symbols were matched as annotated on the platform and in multiMiR. Overlaps were visualized as Venn diagrams using the ggvenn (version 0.1.19) R package.

### 2.4. Animal Model and Sample Collection

Male Wistar rats (8 weeks old) were obtained from the Trakya University Laboratory Animal Research Unit and maintained under standard conditions (22 ± 1 °C, 55% relative humidity, 12 h light/dark cycle, no more than three animals per cage, and ad libitum chow and water). All procedures were approved by the Trakya University Local Animal Ethics Committee on 27 March 2024 (protocol no. TÜHADYEK-2024/12) and followed institutional guidelines. Sample size was determined a priori using effect size estimates (Cohen’s d = 1.6) from previous studies [35,36,37], indicating that 8 animals per group (total *n* = 16) would provide 80% power at α = 0.05 to detect differences in biochemical and expression measures. Animals were randomly assigned to control (*n* = 8) or AMI (*n* = 8) groups using the sample function in R. Myocardial injury was induced by subcutaneous injection of isoproterenol (85 mg/kg in 0.9% NaCl) once daily for two days, a standard model of catecholamine-induced myocardial injury. Sustained β-adrenergic stimulation at this dose produces myocyte necrosis with oxidative and calcium-overload components, distributed diffusely and predominantly subendocardially rather than confined to the territory of an occluded vessel. The model is therefore used here as an independent injury context in which to examine the prioritized transcripts, and not as a reproduction of the coronary-ligation infarction from which the discovery datasets derive. Controls received saline. One AMI animal died during the isoproterenol protocol, so biochemical analyses included 8 control and 7 AMI animals. No analgesics were administered during the isoproterenol protocol, as the model involves no surgical intervention and subcutaneous injection was not expected to cause sustained pain beyond brief, mild discomfort. Animals were observed before and after each injection and at least twice daily between injections for signs of pain or distress. Predefined humane endpoints were severe respiratory distress, inability to stand, and markedly reduced responsiveness to stimuli. Any animal reaching an endpoint was to be euthanized immediately. Apart from these endpoints, no further inclusion or exclusion criteria were applied, and all animals completing the protocol were included in the analyses. At the end of the protocol, animals were anesthetized with intramuscular xylazine (10 mg/kg) and intraperitoneal ketamine (50 mg/kg) and were euthanized by exsanguination via cardiac puncture. Hearts were then excised, rinsed in cold PBS, and snap-frozen at −80 °C for RNA analyses. Serum was obtained by clotting at room temperature for 20 min, centrifugation at 3000 rpm for 20 min, and stored at −80 °C for biomarker assays. The study was designed in accordance with the 3Rs. The discovery phase was conducted entirely on publicly available transcriptomic datasets, so that new animal experiments were restricted to targeted validation of a small, prespecified set of transcripts (replacement). Group size was fixed a priori by power calculation at the minimum required to detect the expected effect, and the subset used for RT-qPCR was drawn from the same cohort rather than from additional animals (reduction). Animals were housed under standard conditions with ad libitum access to food and water, and all terminal procedures were performed under general anesthesia (refinement).

### 2.5. Biomarker Assays

To confirm the presence of cardiac injury, serum levels of creatine kinase-MB (CK-MB) and cardiac troponin-I (cTn-I) were measured using double-antibody sandwich ELISA kits (Btlab, Cat. No. E0311Ra and E0639Ra). Frozen aliquots were thawed at room temperature, gently mixed, and assayed in accordance with the manufacturer’s instructions. Absorbance was read at 450 nm using a microplate reader (AMR-100, Allsheng), and concentrations (ng/mL) were calculated from standard curves provided with the kits.

### 2.6. mRNA Expression by RT-qPCR

RT-qPCR was used to measure transcript levels of candidate genes identified through integrative analysis, on a randomly selected subset of four animals per group for exploratory directional validation of the bioinformatic predictions. The mRNA and the miRNA panels were run on separate randomly selected subsets of the model cohort. The two panels are therefore not paired within animals, and no within-animal association between miRNA and mRNA levels was examined. Each reaction was run in duplicate, and agreement between the two technical replicates of every assay is reported. Left ventricular tissue samples were pulverized under liquid nitrogen and homogenized (DLAB D-160). Total RNA was extracted using the PureLink™ RNA Mini Kit (Thermo Fisher Scientific, Waltham, MA, USA) according to the manufacturer’s instructions. RNA quantity and purity were assessed using a Tecan Infinite M1000 Pro microplate reader, and samples meeting standard purity criteria (A260/280 ≥ 1.8) were used for downstream cDNA synthesis. cDNA was synthesized with the High-Capacity cDNA Reverse Transcription Kit (Thermo Fisher Scientific, Waltham, MA, USA). RT-qPCR was carried out on a 7500 Fast Real-Time PCR System (Applied Biosystems, Foster City, CA, USA) using SYBR Select Master Mix and gene-specific primers. Primer sequences are provided, and primer specificity was confirmed by single-peak melt-curve analysis. Relative expression was calculated by the 2^−ΔΔCt^ method with Gapdh as the reference gene and reported as fold-change relative to controls.

### 2.7. miRNA Expression by RT-qPCR

Total RNA, including small RNAs, was extracted from left ventricular tissue using the mirVana™ miRNA Isolation Kit (Thermo Fisher Scientific, Waltham, MA, USA) following the manufacturer’s protocol. RNA purity and concentration were measured on a Tecan Infinite M1000 Pro microplate reader. cDNA synthesis for target miRNAs employed the TaqMan™ MicroRNA Reverse Transcription Kit with gene-specific stem-loop primers. Quantification was performed in duplicate on a 7500 Fast Real-Time PCR System (Applied Biosystems) using TaqMan™ Universal PCR Master Mix and miRNA-specific probe assays (Thermo Fisher Scientific, Waltham, MA, USA). The assays were 000157 (hsa-miR-214), 002222 (hsa-miR-1), and 002247 (hsa-miR-133b) for the three target miRNAs, and 001973 for U6 snRNA. No assay-specific cross-reactivity data were available for these assays. Relative expression was calculated by the 2^−ΔΔCt^ method using U6 snRNA as the endogenous control. Validation compared AMI and control groups, with results expressed as fold-change relative to controls.

### 2.8. Statistical Analysis

For the microarray differential-expression analyses, group comparisons used the moderated t-statistics of limma described in Section 2.2, and *p*-values were adjusted for false discovery rate using the Benjamini–Hochberg procedure. Statistical significance was defined as adjusted *p* < 0.05.

The continuous outcomes, namely ELISA concentrations of CK-MB and cTn-I and RT-qPCR relative expression values calculated using the 2^−ΔΔCt^ method, were summarized as mean ± standard error of the mean (SEM). Relative expression by the 2^−ΔΔCt^ method is a strictly positive, multiplicative quantity whose logarithm to base 2 is −ΔΔCt. All inferential analyses of the RT-qPCR data were therefore carried out on the ΔΔCt scale, and the resulting estimates and interval limits were back-transformed and are reported as fold changes with 95% confidence intervals. Two considerations motivated this. First, a confidence interval obtained from the t-distribution on the untransformed relative-expression scale is symmetric about the estimate, and for a quantity that cannot be negative such an interval may extend below zero, whereas an interval computed on the ΔΔCt scale and then back-transformed is asymmetric on the fold-change scale and strictly positive. Second, normality of the within-group residuals is rejected on the untransformed relative-expression scale for one transcript (Cyp2e1, *p* = 0.037) but for none on the ΔΔCt scale. Normality was assessed on the analysis scale using the Shapiro–Wilk test applied to the within-group residuals. With eight observations per comparison, this test has limited power, so it was used as the prespecified screening rule rather than as evidence that the residuals are normal. Normality was not rejected for any of the seven transcripts on the ΔΔCt scale (smallest *p* = 0.09, for Fbn1), and group comparisons were accordingly performed with two-sided two-sample Student’s *t*-tests assuming equal variances (six degrees of freedom). The biomarker comparisons (CK-MB and cTn-I, *n* = 8 control and *n* = 7 AMI) were measured on a concentration rather than a ratio scale, and normality was assessed on the untransformed values. CK-MB was consistent with normality (*p* = 0.19) and was compared with a two-sided two-sample Student’s *t*-test, whereas cTn-I departed from it (*p* = 0.042), so the Mann–Whitney U test was applied to that marker. For CK-MB, the confidence interval for the difference in means is from the two-sample t-distribution assuming equal variances. For cTn-I, the Hodges–Lehmann shift estimate and its distribution-free interval are reported. Because both markers separate the two groups completely, the Mann–Whitney *p*-value is the exact permutation value (2/C(15,7) = 0.0003), which is its smallest attainable value at these group sizes. An exact permutation computation was used rather than the normal approximation that the tied values present in each marker would otherwise force. Because each estimate is a back-transformed difference in mean ΔΔCt, it is a ratio of geometric means and differs slightly from the ratio of the arithmetic group means shown. Standardized effect sizes were calculated as Cohen’s d on the ΔΔCt scale, with 95% confidence intervals obtained by inverting the noncentral t-distribution. Because the RT-qPCR subset comprised four animals per group, the smallest standardized difference detectable at 80% power and a two-sided α of 0.05 was determined for a two-sample *t*-test and used to interpret the observed effect sizes. The group size required to detect an effect of the observed magnitude was also estimated as an illustrative projection, because the effect estimates are imprecise with four animals per group. Agreement between the two technical replicates of each reaction was quantified as the absolute difference in raw Ct, and the analysis was repeated using each replicate separately as a sensitivity analysis. All reported confidence intervals quantify sampling uncertainty under the stated analysis assumptions. They do not account for systematic assay error, including the unresolved disagreement between technical replicates. Analyses were conducted in R version 4.5.1, with differential expression analyzed using limma version 3.58.1 and target mapping and intersection performed using multiMiR version 1.32.0. All reported quantities were regenerated from the deposited raw data by the analysis scripts, which a second author independently re-executed and reviewed. Investigators were not blinded to group allocation during outcome measurement or data analysis.

## 3. Results

### 3.1. mRNA and miRNA Differential Expression

In GSE35088, differential expression analysis identified 145 probes corresponding to 143 unique genes meeting the significance threshold (adjusted *p* < 0.05 and |log2FC| ≥ 0.5), of which 112 genes (114 probes) were upregulated, and 31 were downregulated. Log2FC values ranged from −2.0 to +2.3, with several genes showing marked expression shifts. In GSE95855, 35 miRNAs were differentially expressed, comprising 18 upregulated and 17 downregulated. Among these, three miRNAs displayed the most pronounced fold-changes. rno-miR-214-3p was strongly upregulated (adjusted *p* = 0.003, log2FC = 3.22), whereas rno-miR-1b (adjusted *p* = 0.020, log2FC = −2.56) and rno-miR-133b-3p (adjusted *p* = 0.039, log2FC = −2.91) were significantly downregulated. These miRNAs have been previously implicated in cardiac biology, and their altered expression in this dataset highlights them as candidates for further investigation in AMI. Differential expression results are visualized with volcano plots for both datasets (Figure 1A,B).

### 3.2. miRNA–mRNA Interaction Analysis

The 35 differentially expressed miRNAs were mapped to predicted mRNA targets using the multiMiR database, yielding 23,562 unique miRNA–gene pairs (52,203 records across the four prediction tables with rat data), corresponding to 5357 unique predicted targets of the upregulated and 5222 of the downregulated miRNAs, of which 2391 were predicted targets of both. Intersecting these pooled target sets with the differentially expressed mRNAs from GSE35088, in the same direction and in the canonical inverse direction, identified the overlapping genes described in Section 3.3. These overlaps provided the basis for prioritizing directionally concordant candidate sets. They do not by themselves indicate regulation.

### 3.3. Directionally Concordant miRNA–mRNA Candidate Sets

Intersecting the pooled predicted targets of the 18 upregulated miRNAs with upregulated mRNAs identified 22 probes (Table 1) corresponding to 21 unique gene symbols (Figure 2A). Although miRNA upregulation is typically expected to suppress target transcripts, this intersection was restricted to upregulated mRNAs, so the concordant direction of the overlapping transcripts follows from the analysis design rather than from observation. Applying the prioritization criteria described in the Methods, the three genes highlighted as the most robust candidates were Fbn1 (log2FC = 0.95, adjusted *p* = 0.0055), Col3a1 (log2FC = 0.95, adjusted *p* = 0.0188), and Hamp (log2FC = 1.33, adjusted *p* = 0.021). The upregulated miRNAs predicting each gene are listed in Table S4. Fbn1 is a predicted target of miR-214-3p (MicroCosm), whereas Col3a1 (miR-195-5p) and Hamp (let-7b-5p, let-7c-5p) are predicted targets of other upregulated miRNAs and not of miR-214-3p in the queried output.

Conversely, intersecting the pooled predicted targets of the 17 downregulated miRNAs with downregulated mRNAs yielded two overlapping genes (Table 2, Figure 2B), an overlap not significantly different from that expected under independence (Table S5). Among these, Cyp2e1 (log2FC = −0.905, adjusted *p* < 0.001) passed the |log2FC| > 0.9 filter and was prioritized for its established role in xenobiotic metabolism and oxidative stress responses. This intersection was not significantly enriched (Table S5), and Cyp2e1 was therefore prioritized on its own differential expression, which ranks third among the 31 downregulated transcripts by both adjusted *p*-value and fold change. In the queried multiMiR output, Cyp2e1 is a predicted target of miR-212-3p and not of miR-1b or miR-133b-3p (Table S4).

Canonical inverse-direction intersections. For comparison, the canonical intersections were computed with the identical query and gene sets (Figure 2C,D, Tables S2 and S3). Predicted targets of the 17 downregulated miRNAs overlapped with 28 upregulated mRNAs (29 probes, Table S2); among them, the extracellular matrix and TGF-β-pathway transcripts Col3a1, Col4a1, Col5a2, Eln, Lox, Loxl1, Nid2, Tgfb2, Tgfb3, Ctgf, Igfbp7 and Fbn1. Twelve of the 28 were predicted targets of miR-29b-3p and miR-29c-3p, and Col3a1 was predicted in all four prediction databases. Predicted targets of the 18 upregulated miRNAs overlapped with only four downregulated mRNAs, Hdac2, Card9, Scn4b, and Sgca (Table S3), each from a single database. Relative to the overlap expected by chance (Table S5), the canonical up-mRNA intersection was enriched 2.6-fold (28 observed vs. 11.0 expected, one-sided Fisher exact test *p* < 0.001), with the excess concentrated in miR-29b-3p and miR-29c-3p (12 observed against 2.1 and 2.0 expected, Benjamini–Hochberg-adjusted *p* < 0.001, Table S6). The same-direction up-mRNA intersection was enriched 1.9-fold (21 vs. 11.1, *p* = 0.003), and neither intersection involving downregulated mRNAs was significantly enriched (2 vs. 3.0 with *p* = 0.82, and 4 vs. 3.1 with *p* = 0.37). These enrichments were not adjusted for differences in 3′ UTR length or prediction coverage between gene sets. Col3a1 and Fbn1 appear in both the same-direction and the canonical sets, whereas Hamp and Cyp2e1 appear only in the same-direction set, and miR-214-3p has no predicted targets among the downregulated mRNAs.

### 3.4. Experimental Examination of the Candidate Sets

Four mRNAs (Fbn1, Col3a1, Hamp, Cyp2e1) and three miRNAs (rno-miR-214-3p, rno-miR-1b, rno-miR-133b-3p) were selected for experimental validation. RT-qPCR performed on cardiac tissue from the rat isoproterenol model reproduced the direction of change in all seven prioritized molecules, with statistical support for five (miR-214-3p, miR-1b, miR-133b-3p, Col3a1 and Cyp2e1). Hamp increased without statistical support, and Fbn1 showed no statistically supported change (Section 3.5 and Section 3.6).

### 3.5. Biochemical Confirmation of Myocardial Injury

To confirm successful induction of myocardial injury, serum CK-MB and cTn-I were measured (Figure 3A,B). Compared with controls, CK-MB was significantly higher in the AMI group (5.093 ± 0.146 ng/mL in 8 controls vs. 9.261 ± 0.530 ng/mL in 7 AMI animals, difference +4.17 ng/mL, 95% CI +3.05 to +5.29, *p* < 0.001). cTn-I levels were likewise elevated (1.420 ± 0.160 ng/mL in controls vs. 2.933 ± 0.045 ng/mL in AMI animals, shift +1.57 ng/mL (Hodges–Lehmann), 95% CI +1.22 to +1.80, *p* < 0.001), confirming myocardial injury in the model. The two groups did not overlap for either marker. Every AMI animal exceeded every control animal, so the exact Mann–Whitney U test attains its smallest attainable two-sided *p*-value at these group sizes (*p* = 0.0003, Section 2.8).

### 3.6. Bioinformatic–Experimental Concordance

To assess concordance between computational predictions and experimental results, three miRNAs (rno-miR-214-3p, rno-miR-1b, and rno-miR-133b-3p; Figure 4A–C) and four mRNAs (Fbn1, Col3a1, Hamp and Cyp2e1; Figure 5A–D) were quantified by RT-qPCR in rat hearts (*n* = 4 per group). Relative expression was calculated using the 2^−ΔΔCt^ method. Given the small sample size, results are considered exploratory and focus on directional concordance. miR-214-3p was significantly upregulated (2.39 ± 0.11 in AMI vs. 1.05 ± 0.19 in controls, fold change 2.38, 95% CI 1.54–3.67, log2FC = 1.25, *p* = 0.003). Hamp increased without statistical support (2.92 ± 1.05 in AMI vs. 1.25 ± 0.50 in controls, fold change 2.48, 95% CI 0.75–8.21, log2FC = 1.31, *p* = 0.113), the direction matching the microarray data. Both miR-1b and miR-133b-3p were significantly downregulated, miR-1b with a fold change of 0.50 (95% CI 0.37–0.67, log2FC = −1.01, *p* = 0.001) and miR-133b-3p with a fold change of 0.32 (95% CI 0.16–0.66, log2FC = −1.63, *p* = 0.008). Cyp2e1, prioritized alongside these miRNAs, was also strongly reduced (fold change 0.039, 95% CI 0.007–0.21, log2FC = −4.69, *p* = 0.003), in line with array findings (adjusted *p* < 0.001, log2FC = −0.91). Among structural genes, Fbn1 showed no statistically supported change (fold change 1.10, 95% CI 0.29–4.14, log2FC = 0.14, *p* = 0.860), whereas Col3a1 was significantly upregulated (fold change 1.76, 95% CI 1.20–2.58, log2FC = 0.82, *p* = 0.011). Overall, five of the seven prioritized molecules (miR-214-3p, miR-1b, miR-133b-3p, Col3a1 and Cyp2e1) showed statistically supported changes in the same direction as in the discovery datasets, that is, directionally concordant expression changes observed in an independent injury model. Hamp increased without statistical support, and Fbn1 showed no statistically supported change. Among the seven measured molecules, only Fbn1 paired with miR-133b-3p, a predicted regulator of Fbn1 in one database (ElMMo), has the sign pattern that canonical de-repression would produce, and the Fbn1 interval (0.29 to 4.14) is too wide for that pattern to be informative. With four animals per group, the smallest standardized difference detectable at 80% power corresponded to d = 2.4. Fbn1 (d = 0.13) and Hamp (d = 1.31) showed the smallest standardized effects of the seven transcripts and fell below this threshold. Their fold-change confidence intervals spanned both decreases and several-fold increases (Fbn1 0.29 to 4.14 and Hamp 0.75 to 8.21) (Table S8). Agreement between the two technical replicates was close for the reference assays, for Hamp and for all three miRNA assays (median absolute difference ≤ 0.9 raw Ct cycles) but poor for the Fbn1, Col3a1 and Cyp2e1 assays (median 2.5 to 2.9 cycles), and was poorer in the AMI group than in controls (Table S10). Analyzed separately, each replicate reproduces the direction and the statistical support of Cyp2e1 and of all three miRNAs, whereas the estimates for Fbn1, Hamp and Col3a1 differ substantially between replicates, and for Fbn1 and Col3a1 the two replicates place the estimate on opposite sides of no change (Table S9). Which replicate lies closer to the true value cannot be determined from these data, so the direction of the resulting error in the averaged estimate is unknown. The magnitudes reported for Fbn1, Hamp and Col3a1 are therefore provisional; the significant increase in Col3a1 rests on an assay whose replicates did not agree, and the absence of statistical support for Fbn1 and Hamp cannot be read as evidence that these transcripts did not change.

## 4. Discussion

This study integrated bioinformatics and experimental validation to define directionally concordant miRNA–mRNA candidate sets in AMI. Differential expression analysis identified 143 mRNAs and 35 miRNAs altered after infarction, indicating extensive remodeling. miR-214-3p was strongly upregulated, whereas miR-1b and miR-133b-3p were downregulated. Overlap of predicted targets with differentially expressed mRNAs yielded the four prioritized genes Fbn1, Col3a1, Hamp, and Cyp2e1. RT-qPCR in an independent isoproterenol model showed directionally concordant, statistically supported changes for five of the seven prioritized molecules (miR-214-3p, miR-1b, miR-133b-3p, Col3a1, Cyp2e1), while Fbn1 and Hamp showed directionally consistent but nonsignificant increases. Because the candidate sets were assembled by direction, this concordance does not bear on regulatory relationships between the molecules.

The increase in miR-214-3p observed in our dataset is consistent with prior reports showing miR-214 induction in ischemic injury and heart failure models, where it mitigates calcium overload and suppresses pro-apoptotic pathways [38]. Mechanistically, miR-214 represses sodium/calcium exchanger 1 (NCX1) and calcium-dependent death effectors, thereby conferring cardioprotection [38]. In our study, Hamp, a regulator of iron metabolism and inflammatory signaling, increased without statistical support, with the direction matching its microarray increase. Hamp was selected because its discovery-stage change matched the direction of the upregulated miRNAs. Its nonsignificant increase in the independent cohort was directionally consistent but did not confirm differential expression. Hamp is not a predicted target of miR-214-3p in the queried multiMiR output (its predicted regulators among the upregulated miRNAs are let-7b-5p and let-7c-5p, Table S4).

By contrast, both miR-1b and miR-133b-3p were strongly downregulated, consistent with evidence linking suppression of these muscle-enriched miRNAs to hypertrophy and fibrosis [39,40,41]. The miRNA detected here, rno-miR-1b, is annotated in rat and has no separately annotated human counterpart, the corresponding human sequences having been consolidated under miR-1. Its mature form differs from rno-miR-1-3p at a single nucleotide and carries an identical seed. Functional data generated for miR-1 therefore extend to miR-1b only on the basis of shared seed sequence, and the two are not separable by sequence-based target prediction. With that caveat, miR-1 represses ion channel and gap junction proteins such as KCNJ2 and GJA1 in vivo, so that elevated miR-1 is pro-arrhythmic and its reduction has been associated with decreased arrhythmic risk after AMI [39,42]. Similarly, downregulation of miR-133 relieves repression of profibrotic and hypertrophic pathways, contributing to fibroblast activation and extracellular matrix deposition [40,41]. Cyp2e1, a cytochrome P450 enzyme involved in oxidative stress, was suppressed in parallel with miR-1b and miR-133b-3p in both the discovery datasets and the validation cohort. Cyp2e1 is not a predicted target of either miRNA in the queried multiMiR output (Table S4), so this parallel decrease is an observation of directional concordance. Possible explanations include shared upstream regulation and shifts in cell composition in bulk myocardium, neither of which the present design can test.

Col3a1 and Fbn1, encoding key extracellular matrix proteins, increase in the infarcted myocardium, consistent with fibrotic remodeling after MI [43]. In the present cohort, Col3a1 increased with statistical support, whereas Fbn1 showed no statistically supported change (Section 3.6). miR-214-3p is enriched in cardiac fibroblasts and promotes pro-fibrotic phenotypes [44], while hepcidin (Hamp) impairs macrophage-dependent cardiac repair after MI [45]. In parallel, miR-1 and miR-133 modulate oxidative-stress–related survival pathways in cardiomyocytes [40,46], and cardiac Cyp2e1 promotes oxidative stress and apoptosis [47], together reflecting complementary facets of AMI pathology, extracellular-matrix remodeling and oxidative injury. No functional or histological endpoint was measured here. The fibrotic reading of the concordant miR-214-3p and Col3a1 increases, and the oxidative-stress reading of the decreases in miR-1b, miR-133b-3p and Cyp2e1, therefore rest on transcript measurements alone. The pathway descriptions above are drawn from the cited literature on these molecules rather than from endpoints assessed in this study (Limitations).

Taken together, our findings extend, and in several respects differ from, previously reported AMI miRNA networks, recent integrative frameworks for cardiac fibrosis [28], and isolated single-miRNA studies. The closest prior integrative AMI analyses, Wang and Cao [2], who reported 19 differentially expressed miRNAs and over 1000 putative mRNA targets in patient blood, and the recent machine-learning miRNA biomarker panel of Samadishadlou et al. [29], which used sequence-based TargetScan prediction for downstream enrichment, prioritize breadth at the cost of resolution at individual axes. By contrast, we mapped targets using multiMiR predicted interactions, required directional concordance with the mRNA data, and report the canonical inverse-direction intersections alongside (Section 3.3, Tables S2, S3 and S5). The miR-29 family, which dominates that canonical result (Section 3.3), is an established anti-fibrotic regulator of collagen, fibrillin and elastin genes after myocardial infarction [43], and its downregulation in GSE95855 together with the upregulation of its predicted targets in GSE35088 is the pattern expected for canonical repression, although opposite directions of differential expression in two datasets with different animals do not demonstrate it. Because miR-29b/c were not measured in the validation cohort, this axis could not be tested here and is a priority for future work. A stricter alternative would be to restrict mapping to manually curated validated-interaction resources such as miRTarBase (most recently expanded in the 2025 release [48]), which we did not do here. The same-direction requirement narrowed the landscape to two directionally concordant candidate sets, an upregulated set (miR-214-3p with Col3a1, Fbn1 and Hamp) and a downregulated set (miR-1b and miR-133b-3p with Cyp2e1), each of which we additionally examined in vivo. Compared with single-miRNA cardiac studies, namely Aurora et al. [38] (miR-214 in calcium overload and ischemic injury), Sun et al. [44] (miR-214 driving fibroblast proliferation and collagen synthesis after isoproterenol), Yang et al. [49] (miR-214-3p as a fibroblast repressor via NLRC5/TGF-β1–Smad3 in pressure overload), Li et al. [40] (miR-133 in cardiac remodeling), and van Rooij et al. [43] (miR-29 in fibrosis), our work introduces three distinctions. First, the parallel decrease in miR-1b, miR-133b-3p, and Cyp2e1 has not, to our knowledge, been previously reported. It places the suppression of two muscle-enriched miRNAs alongside that of a cytochrome P450 previously linked to cardiac oxidative stress [47], without establishing a functional link between them. This interpretation is biologically reinforced by recent mechanistic evidence showing that Cyp2e1 disrupts OPA1-mediated mitochondrial dynamics and aggravates cardiomyocyte injury in chemotherapeutic and catecholamine-stress settings, with broader relevance across ischemic and hypertrophic models [50]. It also complements earlier transgenic data showing Cyp2e1-driven oxidative stress and apoptosis in the heart [47]. Second, the concordant upregulation of miR-214-3p and Col3a1 in the discovery data and in the validation cohort is consistent with the previously reported pro-fibrotic roles of each molecule [43,44], though neither observation demonstrates a joint function. This observation is distinct from, and does not conflict with, the protective miR-214-3p–NLRC5–TGF-β/Smad3 axis described in pressure overload [49]. It is also distinct from the previously characterized miR-29 [43] and miR-21 [16,17] fibrotic networks. Third, our integrative framework operates within the broader multi-omics paradigm now emphasized for cardiac fibrosis [28]. However, it advances this paradigm by identifying a small number of directionally concordant candidate sets, five of whose seven components changed in the expected direction with statistical support in vivo. These sets bring together molecules previously associated with fibrotic remodeling and with oxidative injury within a single AMI-specific framework, though the present data do not show that they act collectively.

Validated versus predicted interactions. Because the candidate sets were built from predicted interactions, multiMiR’s validated tables were queried for the four prioritized genes in rat, mouse and human, and the records returned were matched to the 35 differentially expressed miRNAs (Table S11). Three of the nine pairings returned by the predicted query have supporting records. Col3a1 pairs with miR-29b-3p, by reporter assay in rat, mouse and human, and with miR-29c-3p by luciferase reporter assay in human, both canonical pairings. Cyp2e1 pairs with miR-212-3p, by luciferase reporter assay in rat (assay types by species in Table S11). The other six are Hamp with let-7b-5p and with let-7c-5p, Col3a1 with miR-195-5p and with miR-1-3p, and Fbn1 with miR-214-3p and with miR-133b-3p, the last two being the only pairings that involve the miRNAs measured here by name. For none of them was a supporting record retrieved from miRTarBase or miRecords in any of the three species. On the same basis, none of miR-214-3p, miR-1b and miR-133b-3p were retrieved as a validated regulator of any of the four genes. Because the assay may not fully distinguish miR-1b from canonical miR-1-3p, the records under that second name also bear on this. The Col3a1 pairing above is unsupported, but FBN1 with miR-1-3p, which the predicted query did not return, carries a weakly supported record (Table S11). Rat coverage of these tables is thin: seven records for these four genes against 435 in humans, so a rat absence is weak evidence of absence. Support beyond sequence-based prediction is therefore concentrated in the miR-29 axis already identified by the canonical analysis.

These findings also have translational implications. Because miRNAs circulate in blood, altered expression patterns can be leveraged as noninvasive biomarkers. Several cardiac-enriched miRNAs, including miR-1, miR-133, miR-208, and miR-499, are elevated in AMI patients and correlate with injury magnitude [51,52,53]. Cardiac-specific miR-208a and muscle-derived miR-499 have shown particular promise as diagnostic markers [52], while early increases in circulating miR-21 demonstrated high discrimination in multicenter cohorts [54]. Our data extend this panel by implicating miR-214-3p and miR-133b-3p, both of which have direct human counterparts, suggesting additional diagnostic or prognostic potential. For miR-1b, the corresponding human analyte would be miR-1, since no separately annotated human miR-1b exists. However, because our validation was limited to cardiac tissue, translational claims remain provisional and require confirmation in circulating samples from clinical cohorts. In a prospective multicentre study of 1155 patients presenting with acute chest pain, the best-performing of six circulating miRNAs reached an area under the curve of 0.76 for acute myocardial infarction, below that of fourth-generation cardiac troponin T at 0.84 and of high-sensitivity troponin T at 0.94, and none of the six added diagnostic value when combined with either assay [55]. In the subgroup presenting within three hours of symptom onset (*n* = 409), where an early marker would be of greatest value, the best-performing miRNA reached an area under the curve of 0.70, and the remaining five did not reach statistical significance, and none of the six predicted long-term mortality. Any biomarker claim for the molecules identified here, diagnostic or prognostic, would therefore have to demonstrate incremental value over high-sensitivity troponin measurement, which that study did not find for any of the six miRNAs it examined. Moreover, the convergence of multiple altered mRNAs with these miRNAs reinforces their potential as therapeutic targets.

Therapeutically, miRNAs are actionable molecules. Antagomirs that inhibit pathogenic miRNAs and mimics that restore protective ones have yielded promising results in experimental models. For example, exogenous augmentation of miR-29 reduces fibrosis [43], while increasing miR-133 suppresses hypertrophy [56]. Conversely, inhibition of detrimental miRNAs such as miR-21 or the miR-15 family reduces infarct size and cell death [16,57]. These observations, together with our data, support continued exploration of miRNA-based interventions in AMI.

This study has several limitations. The transcriptomic datasets came from independent cohorts and platforms, and the lack of paired mRNA–miRNA samples limits causal inference. In addition, the discovery dataset sample sizes were small (5 sham vs. 5 AMI in GSE35088 and 3 sham vs. 3 AMI in GSE95855). Together with the significance thresholds (adjusted *p* < 0.05 and |log2FC| ≥ 0.5 for both mRNAs and miRNAs), these sample sizes determined the resulting numbers of differentially expressed transcripts (143 mRNAs and 35 miRNAs). More liberal thresholds would have produced larger candidate sets but at the cost of increased false-positive risk. The stringent thresholds used here therefore prioritized specificity over sensitivity, which was considered appropriate for downstream targeted validation. Discovery was based on LAD ligation, whereas validation used isoproterenol-induced injury. This cross-model design allowed assessment of whether the individual changes in the identified candidate sets generalized across distinct AMI-related injury contexts. Both models share key pathophysiological features relevant to the candidate sets examined here, including cardiomyocyte death, inflammation, oxidative stress, and fibrotic remodeling [58]. However, LAD ligation produces focal regional ischemia with transmural infarction and a peri-infarct border zone, whereas isoproterenol induces diffuse subendocardial necrosis driven by repeated β-adrenergic activation and reactive oxygen species generation without a primary ischemic component. The small validation subset, however, provided limited sensitivity to differences in the magnitude observed for Hamp and Fbn1. Both transcripts changed in the expected direction, but their standardized effects were the smallest of the seven measured and fell below the difference detectable at this group size (Section 3.6, Table S8). The wide confidence intervals, together with the simultaneous differences between the cohorts, prevent us from distinguishing limited statistical precision from genuine context-dependent differences, or a combination of the two. Because such differences would themselves alter the effect magnitude, the observed effect sizes cannot arbitrate between them. Mechanistic explanations therefore remain plausible but untested. Cardiac hepcidin induction is largely IL-6-driven [45] and may be more strongly activated by focal acute-phase inflammation after LAD ligation than by diffuse catecholamine-induced necrosis, and extracellular matrix remodeling involving Fbn1 may require longer than the two-day isoproterenol exposure used here. A further limitation concerns the technical quality of the mRNA panel. The two technical replicates of the Fbn1, Col3a1 and Cyp2e1 assays disagreed substantially, and analyzed separately, they do not give stable estimates for Fbn1, Hamp or Col3a1 (Section 3.6, Tables S9 and S10). Because these data do not establish which replicate lies closer to the true value, the direction of the resulting error is unknown, and averaging the two cannot be assumed to be conservative. The mRNA fold-change magnitudes are therefore provisional. Col3a1 reached significance on an assay whose two replicates diverged, and the null results for Fbn1 and Hamp are uninformative rather than negative. In contrast, the shared fibrotic and oxidative-stress responses of the two models are consistent with the directionally concordant expression changes observed across the two models for miR-214-3p, Col3a1, miR-1b, miR-133b-3p and Cyp2e1. Because the discovery and validation cohorts differ simultaneously on all the axes set out in Table S7, the study cannot attribute agreement or disagreement between them to any single factor, and the transcripts that changed in the same direction in both are therefore described as showing directionally concordant expression changes across the contexts examined rather than as conserved features of myocardial infarction. Formal external transcriptomic replication of the discovery-stage findings was not performed. Although the cross-laboratory and cross-model design, combining independent LAD ligation datasets with isoproterenol-based RT-qPCR validation, provides a degree of robustness across distinct AMI contexts, independent transcriptomic replication remains necessary.

Direct functional validation of the miRNA–target interactions was also not performed. The proposed targeting relationships were drawn from multiMiR predicted interactions rather than from experimentally validated entries or from reporter or rescue assays conducted in this study. Accordingly, the present work is positioned as an integrative discovery study that establishes correlative miRNA–mRNA relationships rather than causal regulatory mechanisms, and its conclusions are framed in correlative terms throughout. Because the candidate mRNAs were drawn from the pooled predicted-target sets of all differentially expressed miRNAs, and because three of the four prioritized genes are not predicted targets of the three highlighted miRNAs in the queried multiMiR output (Table S4), the candidate sets rest on directional concordance rather than on predicted or demonstrated binding. Same-direction changes do not establish direct targeting, and inverse changes are compatible with direct repression but do not prove it. Because steady-state mRNA levels also reflect transcriptional drive, a net increase does not by itself exclude concurrent repression, a possibility the present data cannot address. Concordant changes can instead arise from shared upstream regulation of fibroblast-enriched transcripts or from cell-composition shifts in bulk tissue, consistent with the established role of miR-214-3p as a pro-fibrotic miRNA enriched in cardiac fibroblasts [44]. The same applies to the downregulated set (miR-1b, miR-133b-3p and Cyp2e1). The canonical inverse-direction analysis (Tables S2 and S3) supplies the comparison. Predicted pairs with opposite directions of differential expression were present in the discovery data, most prominently for miR-29b/c and extracellular-matrix transcripts. The rat prediction tables in multiMiR date from 2009 to 2019 and partly use legacy gene symbols, as does the RatRef-12 platform, on which 35 of the 145 differentially expressed probes carry the “_predicted” suffix. Harmonizing the suffix on both sides enlarges the intersections slightly, without removing any gene and without affecting any transcript selected for RT-qPCR. A re-annotation of both resources to current symbols would be required for a complete mapping. Direct binding and intervention experiments, including dual-luciferase 3′UTR reporter assays, mimic or inhibitor transfection with target rescue in cardiomyocytes or fibroblasts, and in vivo antagomir or mimic studies assessing infarct size, fibrosis, and cardiac function, were beyond the scope of this in silico-led design and represent the logical next step.

Three further limitations concern the cohorts themselves. The experimental validation cohort comprised male rats only, which prevents assessment of sex-specific cardiac responses. Because the mRNA discovery dataset profiled female rats whereas the miRNA dataset and the validation cohort used males, both the direction matching and its examination in vivo compare expression changes across sexes, and any sex-dependent difference in these transcripts would be indistinguishable from the model and timing differences discussed above. Each cohort was sampled at a single post-infarction time point, and the interval differed considerably between them; the discovery datasets having sampled myocardium four weeks after ligation and the validation model two days after the first isoproterenol injection, so temporal expression dynamics could not be evaluated. Finally, bulk left ventricular tissue was measured, which does not resolve cell-type-specific contributions from cardiomyocytes, fibroblasts, endothelial cells, and immune cells. Male animals were studied to reduce estrous-cycle-related variability in miRNA expression within this small exploratory validation cohort. Consequently, the present conclusions should not be generalized to females, and sex-stratified analyses incorporating the modulatory effects of estrogen on cardiovascular miRNA profiles and on myocardial injury and repair represent an important future extension.

The RT-qPCR validation cohort was also small (*n* = 4 per group) and should therefore be considered exploratory, as noted in Section 3.6. This subset was drawn at random from the larger model cohort used for biochemical confirmation and was intended as a targeted examination of the bioinformatically prioritized candidate sets rather than as a primary discovery sample. Its concordance with the independent transcriptomic datasets provides additional, albeit preliminary, support. In addition, rno-miR-1b differs from rno-miR-1-3p at a single nucleotide and shares its seed. The two are carried as separate features on the Agilent-070156 array (GPL22760), and both were differentially expressed in GSE95855, in the same direction (log2FC −2.56 and −2.10), while the RT-qPCR used TaqMan assay 002222, which is directed against the canonical miR-1 sequence. No cross-hybridization or cross-reactivity data are available for either reagent, so neither can be assumed to discriminate fully between the two miRNAs, and the miR-1b measurements reported here may therefore reflect a combined miR-1 family signal. Because expression in the validation cohort was assessed at a single early post-injury time point, the dynamic behavior of these candidate sets across the acute, reparative, and chronic remodeling phases of infarction remains to be characterized. In addition, protein-level confirmation of the reported mRNA changes was not performed, and validation was limited to tissue RNA, making any implications for circulating biomarkers provisional. Because the present study was designed around transcriptomic miRNA–mRNA integration, mRNA abundance was the matched molecular readout for the selected candidate sets. This does not, however, establish corresponding protein-level change, which requires Western blot, immunohistochemistry, or related validation. The analysis of paired peripheral-blood samples is likewise required before circulating-biomarker claims can be established. Clinical studies in acute coronary syndrome indicate the standard such claims must meet. Circulating miR-499 measured in the early phase of non-ST-elevation acute coronary syndrome, for example, predicts long-term major adverse cardiac events [59]. Finally, the present study did not include functional or histological endpoints. Cardiac function was not assessed by echocardiography or invasive hemodynamics, and fibrotic burden was not quantified histologically, for example, by Masson’s trichrome or picrosirius red staining with measurement of collagen volume fraction. Model induction was confirmed biochemically by significant elevations in serum CK-MB and cTn-I (Figure 3), which establishes myocardial injury but does not characterize contractile impairment or the extent of scar formation. The fibrotic interpretation of the miR-214-3p/Col3a1 candidate set therefore rests on transcript-level evidence alone. Although Col3a1 encodes a principal structural component of the post-infarction extracellular matrix and its induction is an established molecular index of fibrotic activation [43], transcript abundance is not a substitute for direct measurement of collagen deposition. Accordingly, the identified candidate sets cannot yet be related to the magnitude of functional deterioration or fibrotic burden in the same animals, and future work pairing echocardiographic indices such as ejection fraction and fractional shortening, and quantitative histological fibrosis assessment, with miRNA–mRNA profiling is required before these molecules can be linked to clinically meaningful outcomes. Future studies should validate direct miRNA-mediated regulation using luciferase 3′UTR reporter assays, miRNA mimic and inhibitor experiments with target rescue in cardiomyocyte cultures, and in vivo antagomir or mimic interventions. Recent work in cardiac fibrosis illustrates this approach, with miR-17-5p shown to attenuate fibrotic remodeling by inhibiting BNIP3-mediated mitochondrial autophagy [60]. Validation in human cohorts and multiple AMI models with paired mRNA–miRNA profiling would further clarify the consistency and context dependence of the identified candidate sets. Recent syntheses of miRNA-based risk stratification and therapeutic development in heart failure outline the translational path that such validation would need to follow [61].

## 5. Conclusions

Our integrative bioinformatic and experimental analyses show that AMI is associated with directionally concordant alterations in specific miRNAs and mRNAs. miR-214-3p was strongly upregulated, while the muscle-enriched miR-1b and miR-133b-3p were downregulated. The mRNAs prioritized alongside them, Col3a1 and Cyp2e1, changed in the same direction with statistical support in the validation cohort, whereas Fbn1 and Hamp did not reach statistical support. The Col3a1 and Cyp2e1 results are not of equal standing. Each technical replicate analyzed separately reproduces the Cyp2e1 decrease, whereas the two replicates of the Col3a1 assay place the estimate on opposite sides of no change. The Col3a1 increase is therefore provisional, pending re-measurement on an assay with acceptable replicate agreement. The seven molecules have previously been implicated in fibrotic extracellular-matrix remodeling (miR-214-3p, Col3a1, Fbn1 [43,44]), iron and inflammatory signaling (Hamp [45]) and oxidative stress (miR-1, miR-133, Cyp2e1 [40,46,47]). The present data do not establish pathway activation or a collective function. No functional or histological endpoint was measured, so the fibrotic reading of the miR-214-3p and Col3a1 changes rests on transcript abundance alone. Because the discovery intersection selected same-direction changes by construction, these candidate sets do not establish regulatory relationships. The two predicted interactions among these molecules both involve Fbn1 (miR-214-3p in the same direction and miR-133b-3p in the inverse direction) and were not tested. The canonical inverse-direction analysis additionally identified 28 upregulated mRNAs as predicted targets of downregulated miRNAs, most prominently Col3a1 and other extracellular-matrix transcripts as predicted targets of the miR-29 family, and Fbn1 as a predicted target of miR-133b-3p. These relationships were not functionally tested here. The candidate sets offer hypotheses for injury and repair and represent potential biomarker signatures. Future work should validate these relationships in circulating samples and clinical cohorts, assess protein endpoints, test the miR-29 axis directly, and explore interventions such as miRNA mimics, inhibitors, or gene-targeted therapies. This work delineates two directionally concordant candidate sets, a downregulated set comprising miR-1b, miR-133b-3p and Cyp2e1 and an upregulated set comprising miR-214-3p and Col3a1, which, to our knowledge, have not previously been described together in AMI, and prioritizes them for mechanistic investigation.

## Figures and Tables

**Figure 1:**
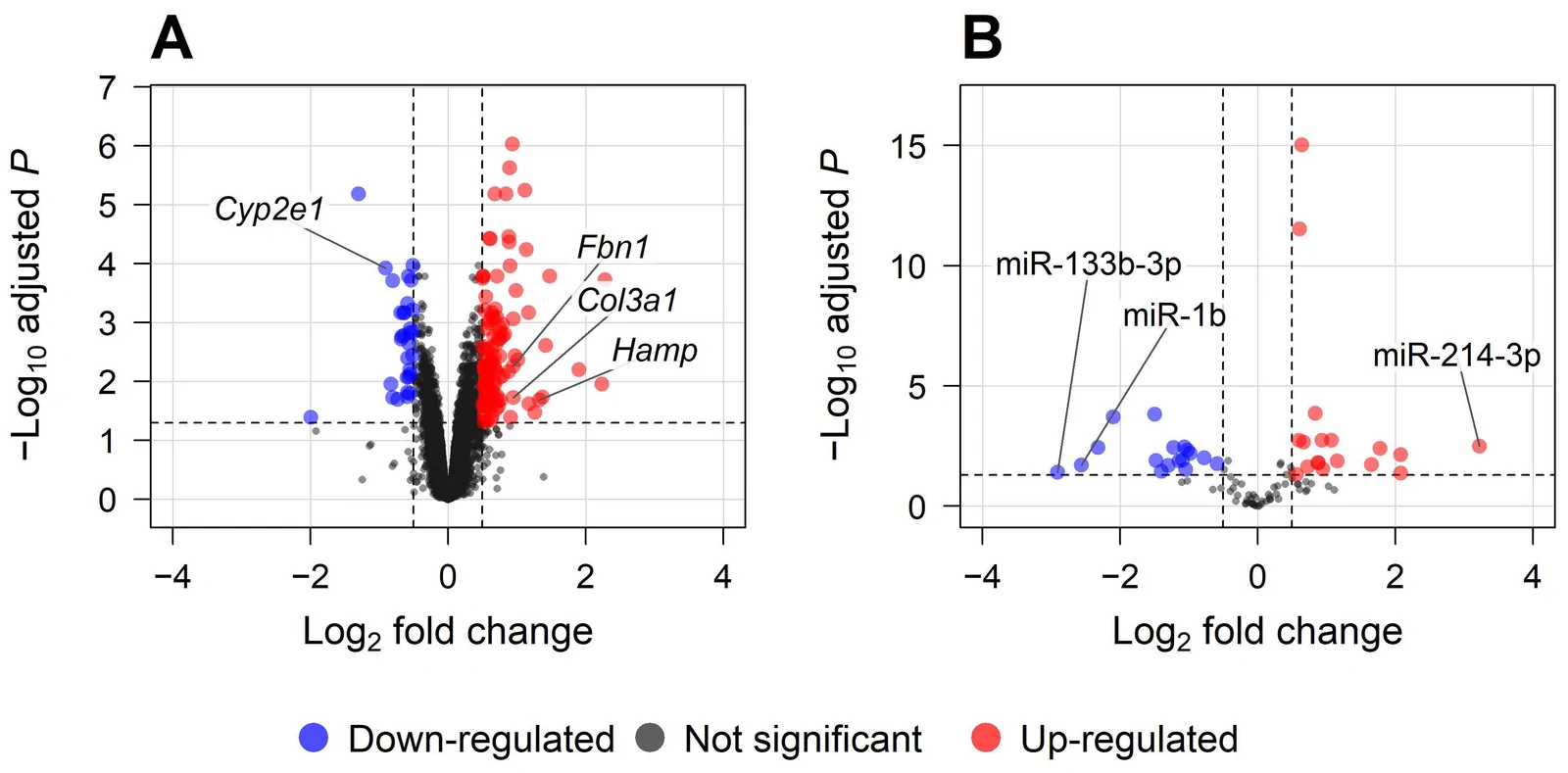
Volcano plots for differential expression in GSE35088 and GSE95855. The *y*-axis shows −log10 of the Benjamini–Hochberg adjusted *p*-value. Dashed lines mark adjusted *p* = 0.05 and |log2FC| = 0.5. (**A**) mRNA probes in GSE35088 (adjusted *p* < 0.05 and |log2FC| ≥ 0.5, with 114 upregulated and 31 downregulated probes, corresponding to 112 and 31 unique genes). The four genes selected for RT-qPCR are labeled. (**B**) miRNAs in GSE95855 (adjusted *p* < 0.05 and |log2FC| ≥ 0.5, with 18 upregulated and 17 downregulated). The three miRNAs with |log2FC| ≥ 2.5 (miR-214-3p, miR-1b, miR-133b-3p), which were carried forward to RT-qPCR, are labeled.

**Figure 2:**
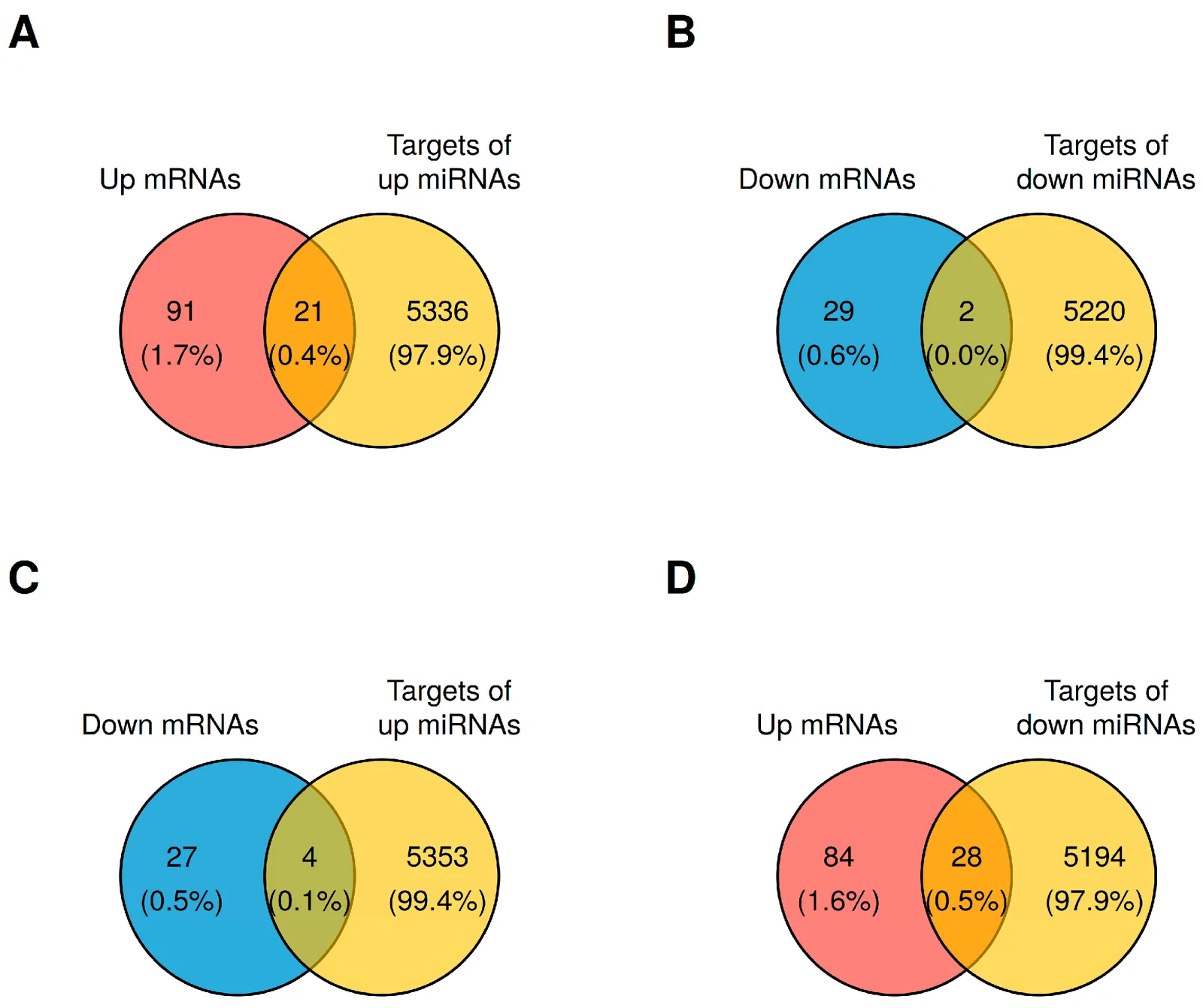
Venn diagrams showing the overlap between predicted mRNA targets of differentially expressed miRNAs (multiMiR, with target sets pooled across all upregulated or all downregulated miRNAs) and differentially expressed mRNAs from GSE35088, for the same-direction and the canonical inverse-direction intersections. (**A**) Upregulated mRNAs versus predicted targets of upregulated miRNAs. (**B**) Downregulated mRNAs versus predicted targets of downregulated miRNAs. (**C**) Downregulated mRNAs versus predicted targets of upregulated miRNAs. (**D**) Upregulated mRNAs versus predicted targets of downregulated miRNAs. Counts are unique gene symbols.

**Figure 3:**
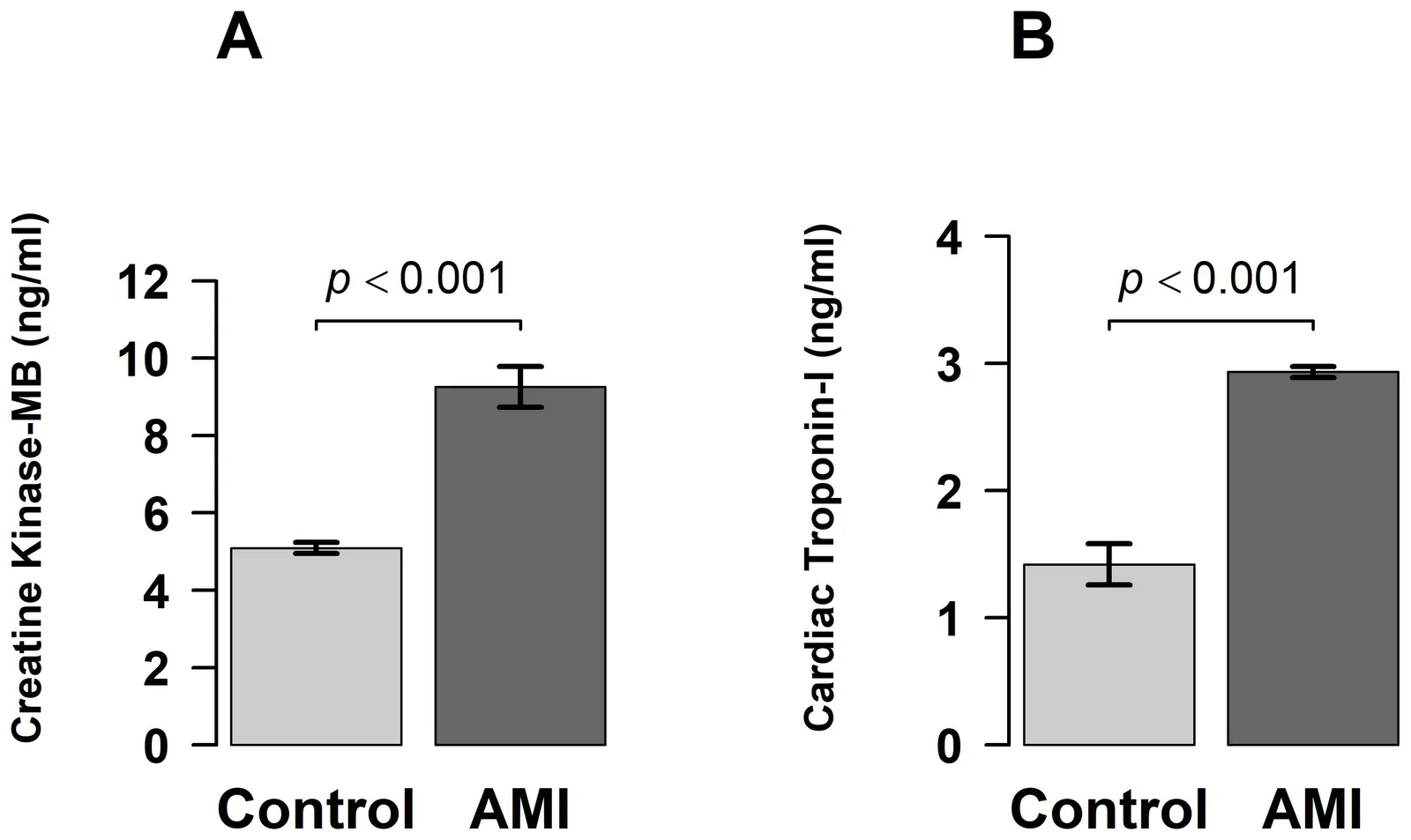
Biochemical confirmation of myocardial injury by (**A**) CK-MB and (**B**) cTn-I serum levels (ng/mL). Bars are mean ± SEM (*n* = 8 control and *n* = 7 AMI).

**Figure 4:**
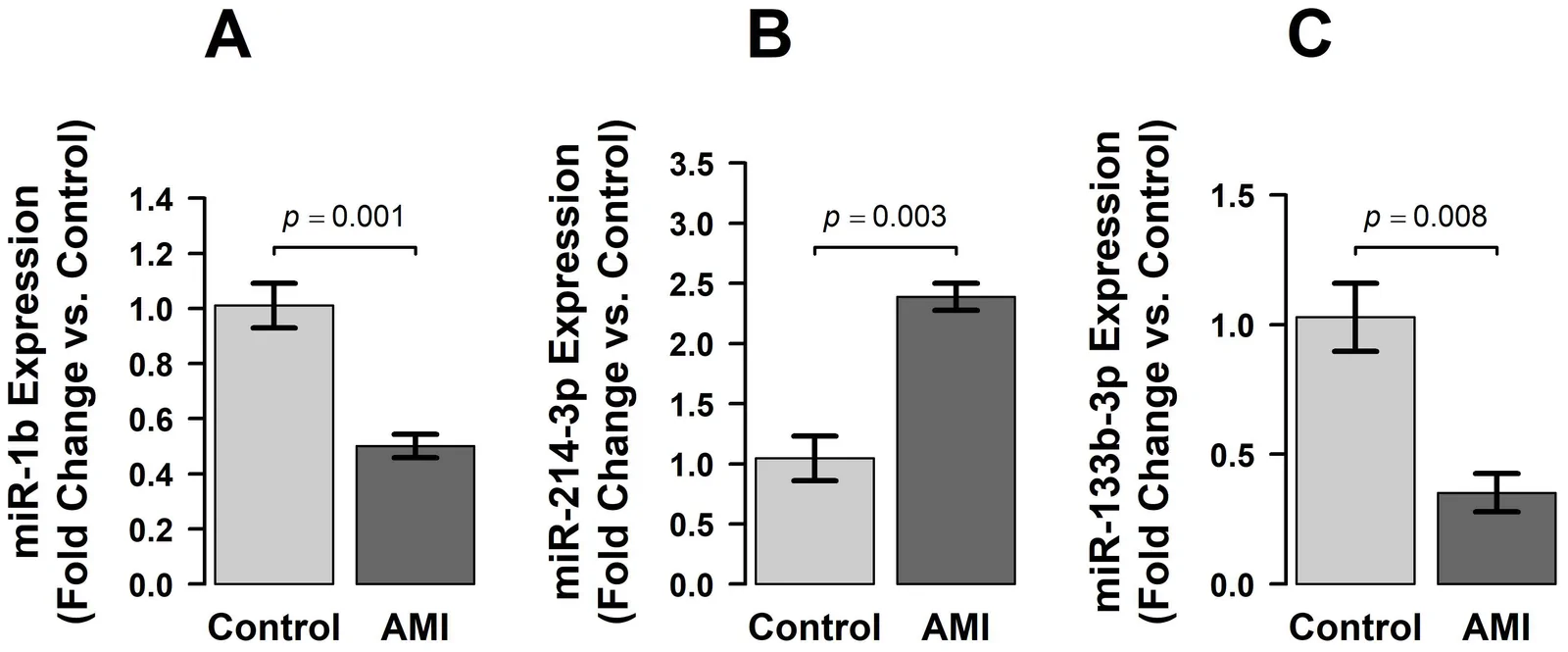
Relative expression by RT-qPCR (normalized to U6 snRNA, 2^−ΔΔCt^ method) for (**A**) miR-1b, (**B**) miR-214-3p, and (**C**) miR-133b-3p in AMI vs. control hearts. Bars are mean ± SEM of relative expression (*n* = 4 per group, each reaction run in duplicate).

**Figure 5:**
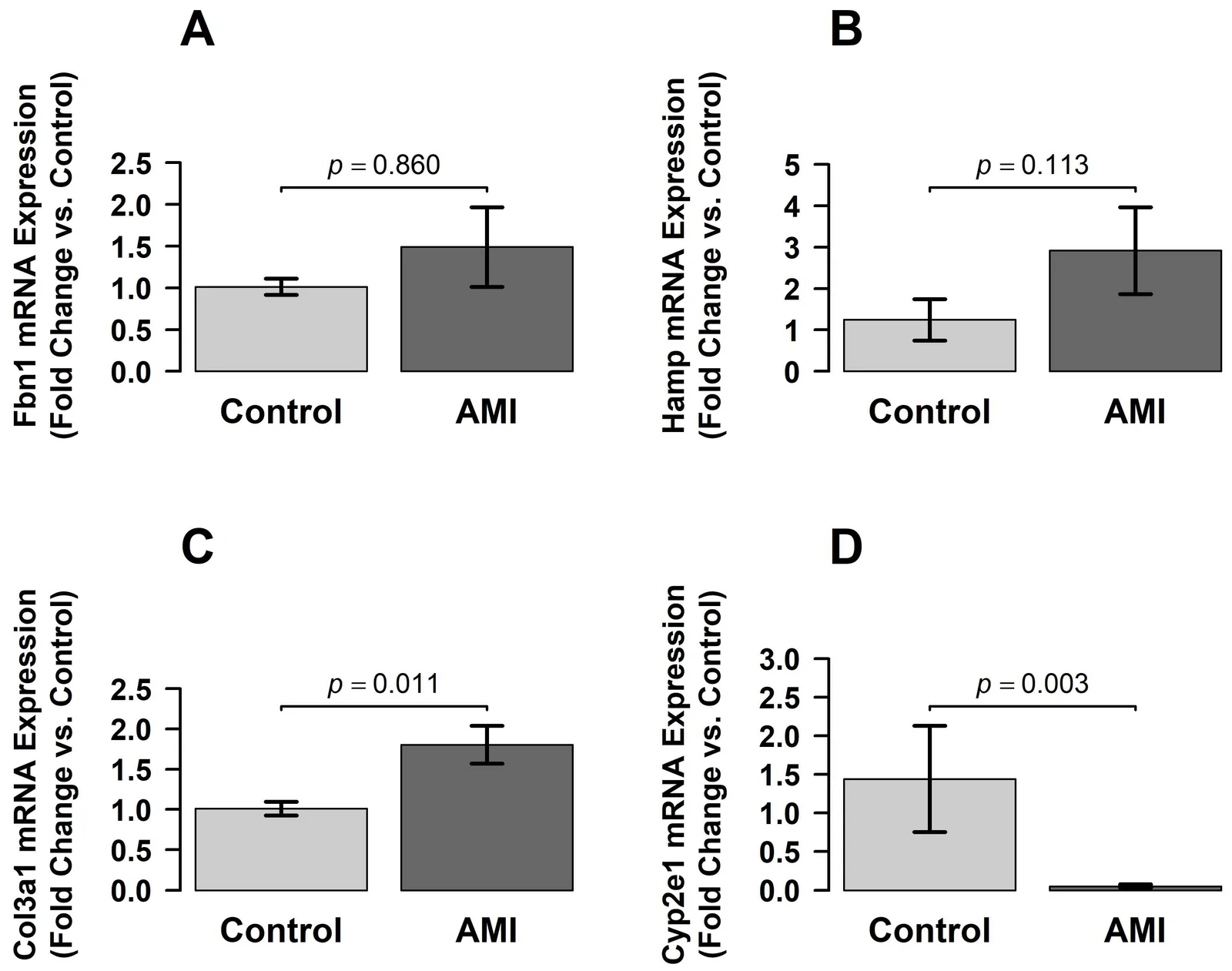
Relative expression by RT-qPCR (normalized to Gapdh, 2^−ΔΔCt^ method) for (**A**) Fbn1, (**B**) Hamp, (**C**) Col3a1, and (**D**) Cyp2e1 in AMI vs. control hearts. Bars are mean ± SEM of relative expression (*n* = 4 per group, each reaction run in duplicate).

**Table 1:** List of the 22 upregulated probes (21 genes) in GSE35088 that overlap with predicted targets of upregulated miRNAs.

ID	Gene Symbol	Adj. *p* Value	t Statistic	B	logFC
ILMN_1357454	Crlf1_predicted	<0.001	11.693	14.837	0.937
ILMN_1376321	Dkk3	<0.001	8.591	9.680	0.601
ILMN_1360318	Col4a1	<0.001	8.523	9.435	0.615
ILMN_1364939	Pam	<0.001	7.541	7.580	0.513
ILMN_1357962	Nbl1	<0.001	7.452	7.427	0.712
ILMN_1349048	Ucp2	<0.001	7.290	7.079	0.505
ILMN_1350770	Scn3b	<0.001	6.391	5.238	0.639
ILMN_1361855	Nid2	0.001	6.023	4.404	0.572
ILMN_1358490	Cdc2a	0.002	5.779	3.864	0.667
ILMN_1369135	Hsd11b1	0.003	5.534	3.310	0.504
ILMN_1367752	Prss23	0.003	5.436	3.087	0.577
ILMN_1360149	Acta1	0.006	5.015	2.126	0.673
ILMN_1359541	Fbn1	0.006	4.999	2.091	0.946
ILMN_1376846	Loxl1	0.007	4.811	1.658	0.882
ILMN_1530405	Loxl1	0.008	4.705	1.410	0.800
ILMN_1358229	Prps2	0.009	4.613	1.187	0.571
ILMN_1650259	Plod2	0.010	4.573	1.108	0.548
ILMN_1355383	Dab2	0.011	4.454	0.834	0.536
ILMN_1350372	Ccnd1	0.011	4.451	0.828	0.539
ILMN_1370763	Serpine2	0.017	4.214	0.268	0.636
ILMN_1354941	Col3a1	0.019	4.118	0.059	0.948
ILMN_1354289	Hamp	0.021	4.048	−0.104	1.330

B is the log-odds statistic from limma, representing the likelihood that the gene is differentially expressed. logFC is the log2 fold change. The suffix _predicted denotes the legacy annotation of the RatRef-12 platform. The t statistic is given for AMI vs. sham.

**Table 2:** List of downregulated mRNAs in GSE35088 that overlap with predicted targets of downregulated miRNAs.

ID	Gene Symbol	Adj. *p* Value	t Statistic	B	logFC
ILMN_1358592	Cyp2e1	<0.001	−7.713	8.018	−0.905
ILMN_1376405	Scn4b	0.002	−5.632	3.532	−0.571

B is the log-odds statistic from limma, representing the likelihood that the gene is differentially expressed. logFC is the log2 fold change. The t statistic is given for AMI vs. sham.

## Data Availability

The datasets analyzed during this study are available in the Gene Expression Omnibus (GEO; https://www.ncbi.nlm.nih.gov/geo/ (accessed on 24 February 2026)), reference numbers GSE35088 and GSE95855. The R scripts and data from this study have been deposited by the authors at https://github.com/selcukorkmaz/AMI_miRNA (accessed on 10 August 2026).

## Supplementary Materials

The following supporting information can be downloaded at: https://www.mdpi.com/article/10.3390/medicina62101860/s1, Table S1: Primer sequences used for RT-qPCR (mRNA targets), with completed primer position ranges; Table S2: Upregulated mRNAs in GSE35088 that are predicted targets of downregulated miRNAs in GSE95855 (canonical inverse-direction intersection; 29 probes, 28 unique genes); Table S3: Downregulated mRNAs in GSE35088 that are predicted targets of upregulated miRNAs in GSE95855 (canonical inverse-direction intersection; 4 probes, 4 unique genes); Table S4: Differentially expressed miRNAs predicting the mRNAs of the same-direction intersections (Table 1 and Table 2 of the main text); Table S5: Observed and expected overlap for the same-direction and canonical intersections between differentially expressed mRNAs (GSE35088) and predicted targets of differentially expressed miRNAs (GSE95855); Table S6: Per-miRNA canonical overlap: predicted targets of each differentially expressed miRNA among the oppositely regulated mRNAs of GSE35088 (miRNAs with at least one overlapping gene); Table S7: Design of the two discovery datasets and of the validation cohort; Table S8: Fold changes with 95% confidence intervals, effect sizes and detectability for the RT-qPCR comparisons (n = 4 per group); Table S9: RT-qPCR results computed from each technical replicate separately and from their mean (n = 4 per group); Table S10: Agreement between the two technical replicates of each RT-qPCR assay, in raw Ct cycles (eight samples per assay); Table S11: Experimentally validated interactions recorded for the four prioritized genes with any of the 35 differentially expressed miRNAs (multiMiR validated tables; rat, mouse and human).
