# Supplementary material for: Integrative Bioinformatics and Exploratory Experimental Validation of Expression Changes in miR-214-3p, miR-1b, miR-133b-3p, and Directionally Concordant mRNAs in a Rat Model of Acute Myocardial Infarction
Source: Medicina (Kaunas). 2026 Sep 25;62(10):1860. doi: 10.3390/medicina62101860 (PMC13706966; doi:10.3390/medicina62101860)
Supplement: Supplementary file 1 [file medicina-62-01860-s001.zip › medicina-4526962-supplementary.pdf]

Supplementary Materials

Supplementary tables for the paper entitled “Integrative Bioinformatics and Exploratory Experimental Validation of Expression Changes in miR-214-3p, miR-1b, miR-133b-3p, and Directionally Concordant mRNAs in a Rat Model of Acute Myocardial Infarction”

Table S1. Primer sequences used for RT-qPCR (mRNA targets).

Gene	Accession No.	Forward primer (5'→3')	Reverse primer (5'→3')	Forward position	Reverse position	Amplicon length (bp)
Fbn1	NM_031825.3	GCTGTGAATGCGACATGGGCTTC	TCTCACACTCGCAGCGGAAGAGG	4619–4641	4734–4756	138
Hamp	NM_053469.2	ATGGCACTAAGCACTCGGAT	AGCCGTAGTCTGTCTCGTCT	18–37	103–122	105
Col3a1	NM_032085.1	TTCCTGGTCCTCCCGGCAAC	GTCCAGCTACTCCAGGGTTTCCA	2740–2759	2843–2865	126
Cyp2e1	NM_031543.2	CGCATCGTGGTCCTGCATGGCTA	GGGAAAACCTCCGCACATCCTT	240–262	381–402	163
Gapdh	NM_017008.4	GCAGCCCAGAACATCATCCCT	CATGCCAGTGAGCTTCCCGTT	673–693	742–762	90

Positions are given relative to the cited RefSeq transcript and span the full length of each primer; the amplicon length is the reverse-primer end position minus the forward-primer start position plus one. Primer specificity was confirmed by single-peak melt-curve analysis (Section 2.6). Two further items of validation are not available for these assays: amplification efficiencies, and confirmation of the amplicons by gel electrophoresis. The $2^{-\Delta\Delta Ct}$ calculation therefore assumes efficiencies close to 100% and comparable between each target and its reference gene; departures from that assumption would bias the fold-change estimates, which is a further reason for treating the magnitudes as provisional (Limitations).

Table S2. Upregulated mRNAs in GSE35088 that are predicted targets of downregulated miRNAs in GSE95855 (canonical inverse-direction intersection; 29 probes, 28 unique genes).

Probe ID	Gene symbol	Adj. p value	t statistic	B	logFC	Predicting DE-miRNAs (no. of databases)
ILMN_1376321	*Dkk3*	<0.001	8.591	9.680	0.601	miR-29b-3p (1); miR-29c-3p (1)
ILMN_1360318	*Col4a1*	<0.001	8.523	9.435	0.615	miR-29b-3p (2); miR-29c-3p (2); let-7g-5p (1)
ILMN_1364939	*Pam*	<0.001	7.541	7.580	0.513	miR-212-3p (1)
ILMN_1357962	*Nbl1*	<0.001	7.452	7.427	0.712	miR-322-5p (1)
ILMN_1350770	*Scn3b*	<0.001	6.391	5.238	0.639	miR-29b-3p (1); miR-29c-3p (1)
ILMN_1364005	*Rnd1*	<0.001	6.233	4.895	0.601	miR-22-3p (1); miR-29b-3p (1); miR-29c-3p (1)
ILMN_1361855	*Nid2*	0.001	6.023	4.404	0.572	miR-29b-3p (1); miR-29c-3p (1); miR-30a-5p (1); miR-30c-5p (1); miR-30d-5p (1); miR-30e-5p (1)
ILMN_1354330	*Maoa*	0.002	5.851	4.019	0.824	miR-322-5p (1)
ILMN_1364113	*Ctgf*	0.002	5.717	3.727	0.754	miR-133b-3p (1)
ILMN_1350180	*Emp1*	0.002	5.711	3.706	0.735	miR-29b-3p (1); miR-29c-3p (1)
ILMN_1358233	*Eln*	0.002	5.573	3.404	1.420	miR-29c-3p (3); miR-29b-3p (2)
ILMN_1357522	*Lrp1*	0.005	5.078	2.270	0.610	miR-107-3p (1)
ILMN_1359541	*Fbn1*	0.006	4.999	2.091	0.946	miR-133b-3p (1)
ILMN_1357041	*Pcp4*	0.006	4.866	1.778	1.900	miR-1-3p (1)
ILMN_1364260	*Anxa2*	0.007	4.816	1.671	0.538	miR-1-3p (3); miR-1b (1); miR-30b-3p (1)
ILMN_1376846	*Loxl1*	0.007	4.811	1.658	0.882	miR-133b-3p (1); miR-22-3p (1)
ILMN_1530405	*Loxl1*	0.008	4.705	1.410	0.800	miR-133b-3p (1); miR-22-3p (1)
ILMN_1376823	*Lox*	0.009	4.653	1.292	0.761	miR-22-3p (1)
ILMN_1373876	*Pmp22*	0.011	4.481	0.897	0.677	miR-29b-3p (3); miR-29c-3p (3)
ILMN_1349776	*RT1-A2*	0.011	4.475	0.858	2.235	miR-30b-3p (1)
ILMN_1350372	*Ccnd1*	0.011	4.451	0.828	0.539	miR-1-3p (1); miR-29b-3p (1); miR-29c-3p (1)
ILMN_1376952	*C1qtnf5*	0.017	4.204	0.180	0.661	miR-22-3p (1)
ILMN_1373682	*Tgfb2*	0.017	4.168	0.161	0.711	miR-133b-3p (1)
ILMN_1354941	*Col3a1*	0.019	4.118	0.059	0.948	miR-29b-3p (4); miR-29c-3p (4); miR-1-3p (1)
ILMN_1360855	*Col5a2*	0.022	4.009	-0.196	0.724	miR-29b-3p (1); miR-29c-3p (1)
ILMN_1352414	*Tgfb3*	0.025	3.903	-0.471	0.532	miR-29b-3p (1); miR-29c-3p (1)
ILMN_1368086	*Igfbp7*	0.038	3.615	-1.103	0.531	miR-322-5p (1)
ILMN_1359657	*Chi3l1*	0.045	3.512	-1.367	0.636	miR-133b-3p (1)
ILMN_1353924	*Anxa1*	0.047	3.483	-1.386	0.572	miR-1-3p (1); miR-30e-5p (1)

Rows are ordered by adjusted p-value. Predicting DE-miRNAs lists every downregulated miRNA with a predicted interaction with the gene (rno- prefix omitted); the number in parentheses is the number of multiMiR prediction databases supporting the pair. t: moderated t statistic (AMI vs sham); B: log-odds of differential expression; logFC: log2 fold change. Genes also present in the same-direction intersection (Table 1): Dkk3, Col4a1, Pam, Nbl1, Scn3b, Nid2, Fbn1, Loxl1, Ccnd1 and Col3a1.

Table S3. Downregulated mRNAs in GSE35088 that are predicted targets of upregulated miRNAs in GSE95855 (canonical inverse-direction intersection; 4 probes, 4 unique genes).

Probe ID	Gene symbol	Adj. p value	t statistic	B	logFC	Predicting DE-miRNAs (no. of databases)
ILMN_1355097	*Hdac2*	<0.001	-6.456	5.362	-0.647	miR-23b-3p (1)
ILMN_1370875	*Card9*	0.002	-5.721	3.734	-0.682	miR-188-5p (1)
ILMN_1376405	*Scn4b*	0.002	-5.632	3.532	-0.571	let-7a-5p (1); let-7b-5p (1); let-7c-5p (1); let-7e-5p (1); let-7i-5p (1); miR-125a-5p (1); miR-125b-5p (1); miR-195-5p (1); miR-497-5p (1)
ILMN_1367518	*Sgca_predicted*	0.009	-4.646	1.278	-0.591	miR-497-5p (1)

Columns as in Table S2. Sgca_predicted: gene symbol as annotated on the RatRef-12 platform and in multiMiR (legacy "_predicted" suffix).

Table S4. Differentially expressed miRNAs predicting the mRNAs of the same-direction intersections (Tables 1 and 2 of the main text).

Set (miRNA / mRNA)	Probe ID	Gene symbol	logFC	Predicting DE-miRNAs (no. of databases)
Up / up (Table 1)	ILMN_1357454	*Crlf1_predicted*	0.937	miR-320-3p (1)
Up / up (Table 1)	ILMN_1376321	*Dkk3*	0.601	let-7a-5p (1); let-7b-5p (1); let-7c-5p (1); let-7e-5p (1); let-7i-5p (1)
Up / up (Table 1)	ILMN_1360318	*Col4a1*	0.615	let-7a-5p (2); let-7b-5p (2); let-7c-5p (2); let-7e-5p (2); let-7i-5p (2); miR-23b-3p (1); miR-320-3p (1)
Up / up (Table 1)	ILMN_1364939	*Pam*	0.513	miR-195-5p (1)
Up / up (Table 1)	ILMN_1357962	*Nbl1*	0.712	miR-497-5p (1)
Up / up (Table 1)	ILMN_1349048	*Ucp2*	0.505	miR-320-3p (1)
Up / up (Table 1)	ILMN_1350770	*Scn3b*	0.639	let-7a-5p (1); let-7b-5p (1); let-7c-5p (1); let-7e-5p (1); let-7i-5p (1)
Up / up (Table 1)	ILMN_1361855	*Nid2*	0.572	let-7a-5p (1); let-7b-5p (1); let-7c-5p (1); let-7e-5p (1); let-7i-5p (1); miR-199a-3p (1)
Up / up (Table 1)	ILMN_1358490	*Cdc2a*	0.667	miR-214-3p (1)
Up / up (Table 1)	ILMN_1369135	*Hsd11b1*	0.504	let-7a-5p (2); let-7e-5p (2); let-7i-5p (2); let-7b-5p (1); let-7c-5p (1)
Up / up (Table 1)	ILMN_1367752	*Prss23*	0.577	miR-320-3p (2)
Up / up (Table 1)	ILMN_1360149	*Acta1*	0.673	let-7a-5p (1); let-7b-5p (1); let-7c-5p (1); let-7e-5p (1); let-7i-5p (1); miR-155-5p (1)
**Up / up (Table 1)**	**ILMN_1359541**	***Fbn1***	**0.946**	**miR-214-3p (1)**
Up / up (Table 1)	ILMN_1376846	*Loxl1*	0.882	miR-125a-5p (1); miR-125b-5p (1); miR-214-3p (1)
Up / up (Table 1)	ILMN_1530405	*Loxl1*	0.800	miR-125a-5p (1); miR-125b-5p (1); miR-214-3p (1)
Up / up (Table 1)	ILMN_1358229	*Prps2*	0.571	miR-214-3p (1)
Up / up (Table 1)	ILMN_1650259	*Plod2*	0.548	miR-199a-3p (1)
Up / up (Table 1)	ILMN_1355383	*Dab2*	0.536	let-7i-5p (1); miR-320-3p (1)
Up / up (Table 1)	ILMN_1350372	*Ccnd1*	0.539	let-7a-5p (1); let-7b-5p (1); let-7c-5p (1); let-7e-5p (1); let-7i-5p (1); miR-195-5p (1); miR-23b-3p (1)
Up / up (Table 1)	ILMN_1370763	*Serpine2*	0.636	miR-199a-3p (2)
**Up / up (Table 1)**	**ILMN_1354941**	***Col3a1***	**0.948**	**miR-195-5p (1)**
**Up / up (Table 1)**	**ILMN_1354289**	***Hamp***	**1.330**	**let-7b-5p (1); let-7c-5p (1)**
**Down / down (Table 2)**	**ILMN_1358592**	***Cyp2e1***	**-0.905**	**miR-212-3p (1)**
Down / down (Table 2)	ILMN_1376405	*Scn4b*	-0.571	miR-107-3p (1); miR-133b-3p (1); miR-29b-3p (1); miR-29c-3p (1); miR-322-5p (1)

Predicting DE-miRNAs lists every miRNA of the stated direction with a predicted interaction with the gene (rno- prefix omitted); the number in parentheses is the number of multiMiR prediction databases supporting the pair. The four genes selected for RT-qPCR are shown in bold: Fbn1 is a predicted target of miR-214-3p, whereas Col3a1 (miR-195-5p), Hamp (let-7b-5p, let-7c-5p) and Cyp2e1 (miR-212-3p) are not predicted targets of miR-214-3p, miR-1b or miR-133b-3p in the queried output (four rat prediction tables, package default cutoff). Crlf1_predicted: gene symbol as annotated on the RatRef-12 platform and in multiMiR (legacy "_predicted" suffix).

Table S5. Observed and expected overlap for the same-direction and canonical intersections between differentially expressed mRNAs (GSE35088) and predicted targets of differentially expressed miRNAs (GSE95855).

Intersection	DEGs (n)	Targets on array (m)	a: DEG and target	b: DEG only	c: target only	d: neither	Expected	Fold	p
A same-direction: up mRNAs x targets of up miRNAs	112	2143	21	91	2122	19440	11.1	1.90	0.003
B same-direction: down mRNAs x targets of down miRNAs	31	2120	2	29	2118	19525	3.0	0.66	0.821
C canonical: down mRNAs x targets of up miRNAs	31	2143	4	27	2139	19504	3.1	1.31	0.368
D canonical: up mRNAs x targets of down miRNAs	112	2120	28	84	2092	19470	11.0	2.56	<0.001

DEGs (n): unique differentially expressed gene symbols in the stated direction. Targets on array (m): unique predicted targets of the pooled up- or downregulated miRNAs that are represented among the N = 21674 unique gene symbols on the GSE35088 array, which served as the universe; all 22,523 probes were tested and eligible for differential-expression selection, and the 117 probes without a symbol, none of them differentially expressed, cannot match a prediction (2,143 of the 5,357 predicted targets of upregulated miRNAs and 2,120 of the 5,222 targets of downregulated miRNAs are represented). a–d are the cells of the 2 × 2 table tested (a + b + c + d = N); Expected = n × m / N; Fold = a / Expected; p: one-sided Fisher exact test (alternative "greater"), reported unadjusted for these four tests; after Bonferroni correction across the four tests, A remains significant ($p = 0.012$) and D remains significant ($p = 1.0 \times 10^{-5}$). Both intersections involving upregulated mRNAs (A and D) show statistically significant enrichment; target-prediction biases, including differences in 3′ UTR length and prediction coverage between gene sets, could contribute to the observed enrichment but were not evaluated here. The excess in D is concentrated in the miR-29 family (Table S6). Enrichment of a pooled target set characterizes the intersection as a whole and does not validate the particular miRNA–mRNA groupings selected for RT-qPCR.

Table S6. Per-miRNA canonical overlap: predicted targets of each differentially expressed miRNA among the oppositely regulated mRNAs of GSE35088 (miRNAs with at least one overlapping gene).

miRNA	Direction	Targets on array	Observed	Expected	Fold	Nominal p	BH-adjusted p	Genes
miR-29c-3p	down	394	12	2.0	5.89	<0.001	<0.001	Ccnd1, Col3a1, Col4a1, Col5a2, Dkk3, Eln, Emp1, Nid2, Pmp22, Rnd1, Scn3b, Tgfb3
miR-29b-3p	down	410	12	2.1	5.66	<0.001	<0.001	Ccnd1, Col3a1, Col4a1, Col5a2, Dkk3, Eln, Emp1, Nid2, Pmp22, Rnd1, Scn3b, Tgfb3
miR-22-3p	down	159	4	0.8	4.87	0.00939	0.11	C1qtnf5, Lox, Loxl1, Rnd1
miR-1-3p	down	286	5	1.5	3.38	0.0165	0.144	Anxa1, Anxa2, Ccnd1, Col3a1, Pcp4
miR-133b-3p	down	330	5	1.7	2.93	0.0285	0.199	Chi3l1, Ctgf, Fbn1, Loxl1, Tgfb2
miR-188-5p	up	94	1	0.1	7.44	0.126	0.654	Card9
miR-497-5p	up	441	2	0.6	3.17	0.131	0.654	Scn4b, Sgca_predicted
miR-30b-3p	down	152	2	0.8	2.55	0.186	0.728	Anxa2, RT1-A2
let-7g-5p	down	40	1	0.2	4.84	0.187	0.728	Col4a1
miR-1b	down	52	1	0.3	3.72	0.236	0.784	Anxa2
miR-23b-3p	up	301	1	0.4	2.32	0.352	0.784	Hdac2
miR-125a-5p	up	317	1	0.5	2.21	0.367	0.784	Scn4b
miR-125b-5p	up	321	1	0.5	2.18	0.371	0.784	Scn4b
miR-195-5p	up	349	1	0.5	2.00	0.396	0.784	Scn4b
miR-322-5p	down	440	3	2.3	1.32	0.398	0.784	Igfbp7, Maoa, Nbl1
let-7a-5p	up	368	1	0.5	1.90	0.412	0.784	Scn4b
let-7c-5p	up	384	1	0.5	1.82	0.426	0.784	Scn4b
let-7i-5p	up	389	1	0.6	1.80	0.43	0.784	Scn4b
let-7b-5p	up	406	1	0.6	1.72	0.444	0.784	Scn4b
let-7e-5p	up	411	1	0.6	1.70	0.448	0.784	Scn4b
miR-212-3p	down	127	1	0.7	1.52	0.483	0.805	Pam
miR-30e-5p	down	385	2	2.0	1.01	0.594	0.945	Anxa1, Nid2
miR-107-3p	down	328	1	1.7	0.59	0.82	1	Lrp1
miR-30d-5p	down	351	1	1.8	0.55	0.84	1	Nid2
miR-30c-5p	down	365	1	1.9	0.53	0.851	1	Nid2
miR-30a-5p	down	368	1	1.9	0.53	0.854	1	Nid2

Universe and test as in Table S5; rows ordered by nominal p-value. Each miRNA was tested against the oppositely regulated differentially expressed genes; BH-adjusted p: Benjamini–Hochberg adjustment applied once across all 35 differentially expressed miRNAs (both directions together). miR-29b-3p and miR-29c-3p share a seed sequence and have identical predicted-target sets among these genes, as do the let-7 family members; seed-sharing miRNAs are therefore not independent tests, which the adjustment does not account for. The following 9 miRNAs had no predicted targets among the oppositely regulated mRNAs and are omitted: miR-126a-3p, miR-214-3p, miR-155-5p, miR-199a-3p, miR-30a-3p, miR-21-5p, miR-320-3p, miR-211-3p, miR-3473.

Table S7. Design of the two discovery datasets and of the validation cohort.

Characteristic	GSE35088 (mRNA discovery)	GSE95855 (miRNA discovery)	Validation cohort
Strain	Sprague-Dawley	Sprague-Dawley	Wistar
Sex	Female	Male	Male
Injury model	LAD ligation	LAD ligation	Isoproterenol, 85 mg/kg s.c. once daily for two days
Time of sampling	Four weeks after ligation	Four weeks after ligation	End of the two-day protocol
Tissue sampled	Non-infarcted (remote) left ventricle	Left ventricle, region not specified in the source study	Whole left ventricle
Group sizes	Sham 5, AMI 5 (T4-treated group not used)	Sham 3, AMI 3	RT-qPCR: control 4, AMI 4; biomarkers: control 8, AMI 7
Readout	Illumina RatRef-12 BeadChip (GPL6101)	Agilent-070156 rat miRNA microarray (GPL22760)	RT-qPCR, $2^{-\Delta\Delta Ct}$
Source	Chen et al. [30]	Wu et al. [31]	Present study

Factors differing between the discovery datasets and the validation cohort: injury model, sampling time, strain, the region of left ventricle sampled, and, relative to GSE35088, sex. Sex also differs between the two discovery datasets, so the direction matching described in Section 2.3 relates mRNA changes measured in female rats to miRNA changes measured in male rats. Because these factors differ simultaneously, agreement or disagreement between the discovery and validation stages cannot be attributed to any single factor.

Table S8. Fold changes with 95% confidence intervals, effect sizes and detectability for the RT-qPCR comparisons (n = 4 per group).

Transcript	Control (mean ± SEM)	AMI (mean ± SEM)	Fold change (95% CI)	log2FC (95% CI)	d (95% CI)	p	n per group for 80% power
miR-1b	1.011 ± 0.081	0.501 ± 0.043	0.50 (0.37–0.67)	−1.01 (−1.45 to −0.58)	−4.00 (−6.57 to −1.36)	0.001	3
miR-214-3p	1.046 ± 0.186	2.388 ± 0.114	2.38 (1.54–3.67)	+1.25 (+0.63 to +1.87)	+3.47 (+1.07 to +5.79)	0.003	3
miR-133b-3p	1.028 ± 0.131	0.352 ± 0.074	0.32 (0.16–0.66)	−1.63 (−2.66 to −0.60)	−2.73 (−4.73 to −0.63)	0.008	4
Fbn1	1.014 ± 0.097	1.489 ± 0.477	1.10 (0.29–4.14)	+0.14 (−1.76 to +2.05)	+0.13 (−1.26 to +1.51)	0.860	922
Hamp	1.247 ± 0.501	2.915 ± 1.047	2.48 (0.75–8.21)	+1.31 (−0.42 to +3.04)	+1.31 (−0.29 to +2.83)	0.113	11
Col3a1	1.012 ± 0.084	1.804 ± 0.234	1.76 (1.20–2.58)	+0.82 (+0.27 to +1.37)	+2.56 (+0.54 to +4.50)	0.011	4
Cyp2e1	1.439 ± 0.688	0.052 ± 0.020	0.039 (0.007–0.21)	−4.69 (−7.12 to −2.25)	−3.33 (−5.59 to −0.99)	0.003	3

Relative expression by the 2^−ΔΔCt method (miRNAs normalized to U6 snRNA, mRNAs to Gapdh), expressed as fold change relative to controls, from the mean of two technical replicates. The Control and AMI columns are arithmetic means of relative expression and correspond to the bars of Figures 4 and 5. Fold change (95% CI): the back-transformed difference in mean ΔΔCt between groups, with the interval from the two-sample t-distribution on six degrees of freedom; because this is a ratio of geometric means it differs slightly from the ratio of the two arithmetic mean columns, most noticeably for Fbn1, whose values include one animal at 0.23-fold. log2FC: the same estimate on the log2 scale. d: Cohen's d on the ΔΔCt scale, computed as the log2 fold change divided by the pooled within-group standard deviation of ΔCt. Because log2 fold change equals mean control ΔCt minus mean AMI ΔCt, the numerator is that contrast and not AMI minus control; d therefore carries the same sign as log2FC. The interval is obtained by inverting the noncentral t-distribution. p: two-sided two-sample Student t-test on the ΔΔCt values. n per group for 80% power: the group size that would be required to detect an effect of the observed magnitude at 80% power and α = 0.05 (two-sided). These projections assume that the observed effects estimate the true effects; with four animals per group the effect estimates are themselves imprecise, and for Fbn1 and Hamp the intervals for d include zero, so the required group size is not bounded above. They are therefore illustrative rather than reliable requirements, and the fold-change intervals are the more informative summary. With the four animals per group available, the smallest detectable standardized difference at 80% power was d = 2.4. All intervals in this table quantify sampling uncertainty under the stated analysis assumptions; they do not account for systematic assay error, including the unresolved disagreement between technical replicates reported in Tables S9 and S10.

Table S9. RT-qPCR results computed from each technical replicate separately and from their mean (n = 4 per group).

Transcript	Replicate 1: fold change (95% CI)	p	Replicate 2: fold change (95% CI)	p	Mean of both replicates: fold change (95% CI)	p
miR-1b	0.62 (0.44–0.87)	0.014	0.40 (0.25–0.63)	0.003	0.50 (0.37–0.67)	0.001
miR-214-3p	2.49 (1.75–3.54)	0.001	2.28 (1.31–3.96)	0.011	2.38 (1.54–3.67)	0.003
miR-133b-3p	0.24 (0.089–0.65)	0.013	0.44 (0.21–0.91)	0.032	0.32 (0.16–0.66)	0.008
Fbn1	6.00 (3.77–9.56)	<0.001	0.20 (0.014–3.03)	0.199	1.10 (0.29–4.14)	0.860
Hamp	4.28 (1.00–18.31)	0.050	1.44 (0.47–4.42)	0.461	2.48 (0.75–8.21)	0.113
Col3a1	7.54 (4.29–13.24)	<0.001	0.41 (0.15–1.14)	0.078	1.76 (1.20–2.58)	0.011
Cyp2e1	0.099 (0.017–0.57)	0.018	0.015 (0.002–0.11)	0.002	0.039 (0.007–0.21)	0.003

Each reaction was run in duplicate. The analysis reported in the main text uses the mean of the two replicates (right-hand columns), as stated in Section 2.6; the two left-hand pairs of columns repeat it using each replicate alone. Fold changes are back-transformed differences in mean ΔΔCt with intervals from the two-sample t-distribution, and p-values are from two-sided two-sample Student t-tests on the ΔΔCt values. The direction and the statistical support of Cyp2e1, miR-1b, miR-214-3p and miR-133b-3p are reproduced by each replicate individually. The estimates for Fbn1, Hamp and Col3a1 are not stable between replicates: for Fbn1 and Col3a1 the two replicates place the estimate on opposite sides of no change. Relative to the first replicate alone, the estimates from the mean of both replicates are lower for the three upregulated transcripts and more negative for Cyp2e1. The Cyp2e1 fold change of 0.099 shown here for the first replicate corresponds to log2FC = –3.34; the submitted manuscript reported –3.87 for the same replicate because it was computed as the logarithm of the ratio of the two arithmetic group means rather than as the difference in mean ΔΔCt (Section 2.8). This is arithmetic and not evidence about accuracy: these data do not establish which replicate lies closer to the true value, so the direction of the resulting error in the averaged estimate is unknown.

Table S10. Agreement between the two technical replicates of each RT-qPCR assay, in raw Ct cycles (eight samples per assay).

Assay	Panel	Median |Δ| (cycles)	Maximum |Δ|	Samples with |Δ| > 1.5 cycles	Mean signed difference, replicate 2 – replicate 1 (all; control; AMI)
Gapdh	mRNA (reference)	0.24	1.12	0	+0.25; +0.58; −0.09
Fbn1	mRNA (target)	2.75	9.42	5	+2.99; +0.88; +5.09
Hamp	mRNA (target)	0.86	1.12	0	+0.41; −0.04; +0.86
Col3a1	mRNA (target)	2.89	5.39	5	+2.57; +0.81; +4.33
Cyp2e1	mRNA (target)	2.51	5.68	6	+2.52; +1.50; +3.53
U6 snRNA	miRNA (reference)	0.07	0.90	0	−0.05; +0.12; −0.22
miR-1b	miRNA (target)	0.26	0.76	0	−0.26; −0.41; −0.11
miR-214-3p	miRNA (target)	0.19	0.63	0	+0.13; +0.23; +0.02
miR-133b-3p	miRNA (target)	0.89	1.21	0	−0.39; +0.20; −0.99

|Δ|: absolute difference between the two technical replicates of the same reaction, in raw Ct cycles; one cycle corresponds to a two-fold difference in estimated template abundance. Agreement was close for both reference assays and for all three miRNA target assays. For the Fbn1, Col3a1 and Cyp2e1 assays the replicates differed by a median of 2.5 to 2.9 cycles, with five or six of the eight samples exceeding 1.5 cycles and a maximum of 9.4 cycles (Fbn1, one AMI animal). The discrepancy is one-directional and group-dependent: the second replicate returned systematically later Ct values for these three target assays (mean +2.5 to +3.0 cycles, $p \leq 0.03$ against no difference), and the shift was larger in the AMI group (+3.5 to +5.1 cycles) than in controls (+0.8 to +1.5), whereas the Gapdh assay measured in the same reactions showed no shift (+0.25 cycles, $p = 0.26$). The mRNA fold-change magnitudes should therefore be read as provisional; the direction-level conclusions for Cyp2e1 and for the three miRNAs are unaffected (Table S9).

Table S11. Experimentally validated interactions recorded for the four prioritized genes with any of the 35 differentially expressed miRNAs (multiMiR validated tables; rat, mouse and human).

Gene	miRNA	Pairing in this study	Validated evidence, by species	Database	PMID
Col3a1	miR-29b-3p	canonical	reporter/blot: human, mouse, rat	miRecords, miRTarBase	18723672
Col3a1	miR-29c-3p	canonical	reporter/blot: human	miRecords, miRTarBase	18390668
Cyp2e1	miR-212-3p	same-direction	reporter/blot: rat	miRTarBase	23920219
Col3a1	let-7b-5p	not returned by the predicted query	reporter/blot: human	miRTarBase	22995917
Col3a1	let-7e-5p	not returned by the predicted query	reporter/blot: mouse	miRTarBase	26272747
Fbn1	miR-1-3p	not returned by the predicted query	proteomics or CLASH only: human	miRTarBase	18668040
Fbn1	miR-125b-5p	not returned by the predicted query	proteomics or CLASH only: human	miRTarBase	23622248
Fbn1	miR-29b-3p	not returned by the predicted query	reporter/blot: human, mouse	miRecords, miRTarBase	18723672
Fbn1	miR-29c-3p	not returned by the predicted query	reporter/blot: human	miRecords, miRTarBase	18390668
Fbn1	miR-30a-5p	not returned by the predicted query	proteomics or CLASH only: human	miRTarBase	18668040

Query: multiMiR with table = “validated” for the four genes, run in rat, mouse and human by validatedInteractions.R, with the records returned matched to the 35 differentially expressed miRNAs used for the predicted mapping. In rat the query was issued over those 35 miRNAs together with the four genes; in mouse and human it was issued for the genes alone, because several rat names have no counterpart under the same name in those species and a prefix-swapped query would have returned an empty result silently. The full output is deposited as data/validated_interactions_by_species.txt and the coverage counts as data/validated_coverage_by_species.txt. miRNA names are given in their rat form; mouse and human records were matched on the species-stripped name, with mmu-miR-1a-3p treated as the mouse form of miR-1-3p. “Pairing in this study” states whether the predicted query returned that gene–miRNA pair, and if so in which direction relation. “Reporter/blot” denotes miRTarBase support type “Functional MTI”, whose experiments include a luciferase reporter assay, immunoblot or Northern blot; “proteomics or CLASH only” denotes “Functional MTI (Weak)”, supported by proteomics, CLASH or microarray without a reporter assay. Only the first PMID is shown where a record cites several. TarBase records are excluded from the counts and their status is reported as unresolved rather than as evidence against an interaction, because a negative result can be genuine and laboratories can disagree. All rows are in the deposited output. Of the nine pairings returned by the predicted query, the six not listed here, Col3a1 with miR-1-3p and with miR-195-5p, Fbn1 with miR-133b-3p and with miR-214-3p, and Hamp with let-7b-5p and with let-7c-5p, returned no supporting record from miRTarBase or miRecords in the rat, mouse or human queries. Four of them returned an unresolved TarBase row (Col3a1 with miR-1-3p in human and mouse, Col3a1 with miR-195-5p in human, Fbn1 with miR-214-3p in human, and Hamp with let-7c-5p in mouse); the remaining two, Fbn1 with miR-133b-3p and Hamp with let-7b-5p, returned no record of any kind. Coverage differs greatly between species: the four genes returned 7 validated records in rat, 177 in mouse and 435 in human, so the absence of a rat record is weak evidence of absence.
